# Dissecting Nucleosome Free Regions by a Segmental Semi-Markov Model

**DOI:** 10.1371/journal.pone.0004721

**Published:** 2009-03-06

**Authors:** Wei Sun, Wei Xie, Feng Xu, Michael Grunstein, Ker-Chau Li

**Affiliations:** 1 Department of Biostatistics, Carolina Center for Genome Science, University of North Carolina, Chapel Hill, North Carolina, United States of America; 2 Department of Genetics, Carolina Center for Genome Science, University of North Carolina, Chapel Hill, North Carolina, United States of America; 3 Department of Biological Chemistry, University of California Los Angeles, Los Angeles, California, United States of America; 4 Department of Statistics, University of California Los Angeles, Los Angeles, California, United States of America; 5 Institute of Statistical Science, Genomics Research Center, Academia Sinica, Taipei, Taiwan; University of East Piedmont, Italy

## Abstract

**Background:**

Nucleosome free regions (NFRs) play important roles in diverse biological processes including gene regulation. A genome-wide quantitative portrait of each individual NFR, with their starting and ending positions, lengths, and degrees of nucleosome depletion is critical for revealing the heterogeneity of gene regulation and chromatin organization. By averaging nucleosome occupancy levels, previous studies have identified the presence of NFRs in the promoter regions across many genes. However, evaluation of the quantitative characteristics of individual NFRs requires an NFR calling method.

**Methodology:**

In this study, we propose a statistical method to identify the patterns of NFRs from a genome-wide measurement of nucleosome occupancy. This method is based on an appropriately designed segmental semi-Markov model, which can capture each NFR pattern and output its quantitative characterizations. Our results show that the majority of the NFRs are located in intergenic regions or promoters with a length of about 400–600bp and varying degrees of nucleosome depletion. Our quantitative NFR mapping allows for an investigation of the relative impacts of transcription machinery and DNA sequence in evicting histones from NFRs. We show that while both factors have significant overall effects, their specific contributions vary across different subtypes of NFRs.

**Conclusion:**

The emphasis of our approach on the variation rather than the consensus of nucleosome free regions sets the tone for enabling the exploration of many subtler dynamic aspects of chromatin biology.

## Introduction

Nucleosomes, the building blocks of chromatin, are critical regulators in many biological processes, such as transcription, DNA repair and replication [1]. The presence of nucleosomes in many occasions hinders the accessibility of the transcriptional machinery to access the underlying DNA. Conversely, nucleosome depletion allows access of transcriptional regulators to DNA sequences [2]–[6]. This underlines the importance of locating nucleosome positions, a goal that has been attained by several groups in yeast [2]–[6] and mammals [7]–[8].

Despite the availability of genome wide nucleosome distribution profiles, several fundamental questions regarding the nature of nucleosome free regions (NFRs) remain unknown. First, it is not clear whether NFRs occur exclusively at the promoter regions. NFRs in non-promoter regions (including coding regions) may have functions that have not been identified. Second, it has been controversial whether histones are depleted only from the promoters of activated genes. Several studies suggested the existence of transcription-independent NFRs at individual promoters [7]–[10]. Finally, the transcriptional machinery and DNA sequence have been shown to be involved in histone eviction [9]–[11], however, they may have distinct effects for different subtypes of NFRs

To investigate the above issues, it is important to bring out the dynamic aspects of NFRs. Due to the complex interplay between gene regulation and chromatin remodeling, the lengths of NFRs may differ from one another. Likewise, the degree of nucleosome depletion (DoND) in each NFR is likely to vary as well. However, while many previous studies have described nucleosome occupancy in quantitative terms, most of them focused on ensemble properties of NFRs. For instance, representative nucleosome occupancy in the promoter regions have been reported by averaging the enrichments of nucleosomes across all genes aligned by the start codons of ORFs or transcription start sites (TSSs) [5], [6]. Although this “representative NFR” reveals a shared pattern of nucleosome depletion for many genes, it also masks characteristics specific for individual NFRs. On the other hand, reports such as Fig. 4 of Lee et al. [6] and Fig. 2 of Whitehouse et al. [14] did indicate length variation among NFRs. However, the location and quantitative features of each individual NFR have not been systematically explored.

In order to examine an individual NFR across the whole genome, an automatic “NFR calling” algorithm is required that can dissect an NFR pattern from a noisy background. Currently the major existing algorithms facilitating the analysis of genome-wide DNA-protein interaction data were adapted from those initially designed for detecting the binding of transcription factors (TFs) [12]–[16]. These algorithms are however inadequate for capturing NFRs for the following reasons: first, as most TFs are sparsely localized across the genome, many algorithms for identifying TF binding sites (TFBSs) are designed under the assumption that TF binding is an uncommon event, thus the majority of array data is considered background noise. However, this assumption becomes problematic for exploring epigenetic events that are often abundant, including the occupancy of nucleosomes and a variety of histone modifications. Second, the signal of TF binding obtained from microarrays typically occurs within a short region and tends to form a sharp “peak” (Supplementary Figure 1a in Supplementary Materials S1). In contrast, the pattern of nucleosome occupancy or the occurrence of histone modifications can be much longer with various lengths (Supplementary Figure 1b in Supplementary Materials S1). Third, while the binding of a TF at a promoter only requires a qualitative description (i.e. presence/absence), quantitative characterizations such as the DoND are essential for NFRs.

To our knowledge, there are only two published algorithms specifically designed for detecting the presence of nucleosomes. Yuan et al. [5] employed a Hidden Markov Model (HMM) to characterize positioned or delocalized nucleosomes. This algorithm was further modified to analyze higher resolution nucleosome occupancy data by Lee et al. [6]. This method only infers the positions of nucleosomes, but does not provide quantitative properties of the NFRs such as DoND. Alternatively, Ozsolak et al. [17] proposed a two-step procedure to detect positioned nucleosomes, consisting mainly of (1) smoothing the raw probe-level data by wavelet decomposition and (2) decomposing the entire chromosome into “peaks” and “troughs” by an edge-detection technique. A peak-to-trough ratio was used to quantify the signal of nucleosome occupancy, which is a conservative measurement since it is estimated using only a few extreme values. As Ozsolak et al. mentioned in their supplementary methods “This method gives a very conservative definition of peak and trough heights and eliminates many false positives”.

**Figure 1:**
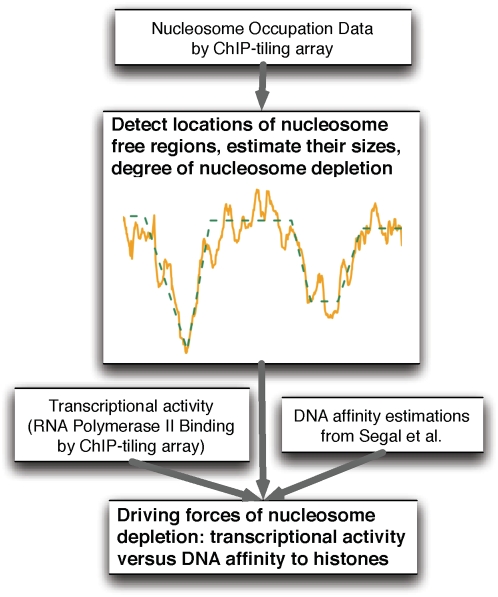
A schematic representation of our study. A flow chart is shown to illustrate the experimental and computational strategies employed in this study. In the central panel, the solid orange line indicates observed histone binding signals and the green dash line indicates the fitted result using our SSMM model.

In this study, we developed an algorithm for capturing complex signal patterns from high-density genome-wide data. This algorithm is based on a segmental semi-Markov model (SSMM) which is an extension of HMM. In addition to identifying desired patterns (e.g., NFR patterns) and capturing their quantitative features, our algorithm also enjoys more flexible model assumptions and higher efficiency compared to regular HMM. A schematic representation of our study is shown in Figure 1, depicting how SSMM is used in characterizing genome-wide NFRs and exploring the driving forces of nucleosome depletion.

## Results

### Data collection and validation

To measure nucleosome occupancy genome wide in yeast using ChIP-chip assays, we isolated yeast nucleosomal DNA by chromatin immunoprecipitation (ChIP) using anti-H3 antibodies and hybridized both immunoprecipitated and input DNA to the Affymetrix *Saccharomyces cerevisiae* Tiling 1.0R Array featuring a 4-bp high resolution (See Methods). Two different H3 antibodies (each with two biological replicates) were utilized which generated highly reproducible results (Pearson correlation R = 0.82 across ∼2.6 millions probes). We used the average of the four replicates for the following analysis.

We first validated our data using previously published nucleosome occupancy data. A coarse-grained version of our data show consistency with previous works including those from Bernstein et al. [2] (R = 0.66, ∼6000 probes), Lee et al. [3] (R = 0.77, ∼12000 probes) and Pokholok et al. [4] (R = 0.62, ∼41000 probes) (Supplementary Tables 1–3 in Supplementary Materials S1). We further compared our data to two recent nucleosome maps at higher resolution [5], [6]. Using an averaging window of 500bp, the correlations between our data and the data from Lee et al. [6] and Yuan et al. are 0.75 and 0.59, respectively. Therefore our data are of high quality and are consistent with published works.

### Nucleosome occupancy levels for various chromosome features

We first examined nucleosome occupancy at various chromosomal features. Previous studies reported relatively low nucleosome occupancy in regions such as promoters and enhancers [1]–[3], [5], [11]. A more comprehensive survey was performed for H2A.Z-containing nucleosomes at different chromosome features [18]. Here we carried out a systematic study for average nucleosome occupancy on all annotated chromosome features, including ORFs, ARSs, rRNAs, tRNAs, snRNAs, snoRNAs, telomeric elements, introns, long terminal repeats and transposons. Specifically, We measure the nucleosome occupancy in one chromosome feature, e.g., ORF, by the log ratio (the ChIP enriched signals versus the signals from genomic control) averaged across all the instances of the chromosome feature, e.g., all individual ORFs. Our findings reveal that ORFs, transposons, rRNAs, telomeres (except the telomeric repeats where there is presumably no histone binding [19]) have higher nucleosome occupancy on average than intergenic regions. In contrast, genes coding for tRNAs and snoRNAs have significantly lower nucleosome occupancy than intergenic regions, presumably due to their intense transcriptional activities [20]. Interestingly, introns and ARSs also have low nucleosome occupancy (Supplementary Table 4 in Supplementary Materials S1).

### NFR identification by SSMM

**Figure 2:**
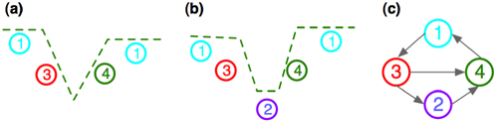
Four states in segmental semi-Markov model. (a) A triangle pattern with multiple states is shown representing the first type of NFR signal observed from tiling array-based ChIP-chip data. (b) A trapezoid pattern with multiple states is shown representing the second type of NFR signal observed from tiling array-based ChIP-chip data. (c) The allowed transitions between any two of the four states in (a) and (b).

In order to examine individual NFR across the whole genome, we developed an automatic “NFR calling” algorithm based on a segmental semi-Markov model (SSMM) to capture the quantitative properties of each NFR. A schematic plot is shown in Figure 2 to illustrate our SSMM, which includes four states. States 1 and 2, represented by two horizontal lines, reflect signals from a nucleosome occupied region (NOR) and an NFR, respectively. These two states have the same shape, but different state duration and transition probabilities. State 3, represented by a line with negative slope, models the transition from the NOR to the NFR; and state 4, represented by a line with positive slope, models the transition from the NFR to the NOR. Initial inspection of the nucleosome occupancy data revealed two types of NFR patterns: triangles (Figure 2(a)) and trapezoids (Figure 2(b)). A triangle or trapezoid pattern corresponds to the path: 1→3→4→1 or 1→3→2→4→1, respectively (Figure 2(c)). Therefore our SSMM algorithm can be considered as a stochastic curve-fitting algorithm to capture both triangular and trapezoidal patterns.

**Figure 3:**
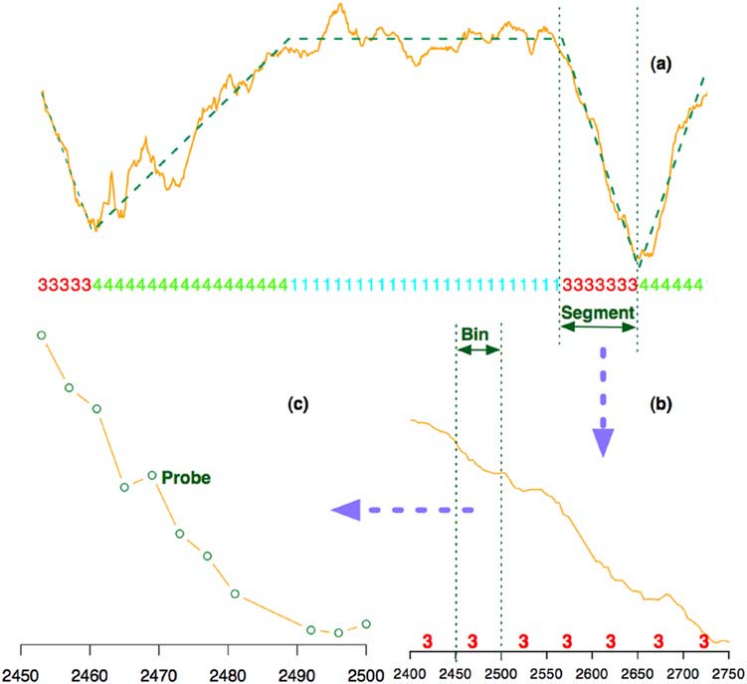
The organization of our segmental semi-Markov model. (a) The yellow solid line represents observed data and the green dash line indicates the model fitting by SSMM. The state of each bin is labeled by numbers 1, 2, 3, or 4 as shown at the bottom. A magnified seven-bin long segment between the two vertical dotted lines is shown in part (b). A single 50-bp bin from segment in (b) containing 11 probes is shown in (c). It can be seen that the distances between adjacent probes are not constant.

To implement this SSMM algorithm, we organized the data into three hierarchical levels: probes, bins, and segments (Figure 3). We first grouped probes within a 50-bp window into a “bin”. Then “segments” were constructed from one or several “bins” so that all the probes within a segment are emitted from the same hidden state. The emission probability (i.e., the probability to observe this segment given the underlying state) was calculated based on linear model fitting. This design has greatly improved the efficiency and robustness of our method (see Methods section for details).

**Figure 4:**
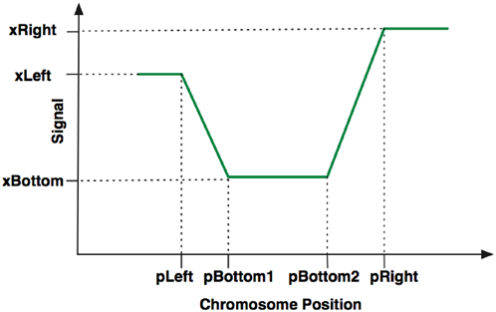
Quantitative characterizations of NFRs. This figure shows how to calculate quantitative features of NFRs based on a trapezoid pattern. Four quantities can be estimated as follows: location, 0.5*(pBottom1+pBottom2); “absolute depletion”, xBottom; “relative depletion”, min(xLeft, xRight)-xBottom; range, [0.5(pLeft+pBottom1), 0.5(pRight+pBottom2)]. The triangle pattern is just one special case of trapezoid pattern, with pBottom1 = pBottom2.

SSMM fitting enables us to derive four quantitative features of each NFR: (1) the location, (2) the length, (3) the absolute DoND level (“absolute depletion”), and (4) the relative DoND level (“relative depletion”). As a triangle pattern is a special case of a trapezoid pattern with its bottom degenerated to one point, we only describe how to obtain the quantitative features for trapezoids. As illustrated in Figure 4, we define the location of an NFR as the position of the mid-point of its bottom. The length of an NFR is defined as the horizontal distance between the mid-points of two opposite sides. The “absolute depletion” (“*A”* for short) measures the signal level (log ratio) at the bottom. The “relative depletion” (“*R”* for short) measures the signal decrease in an NFR compared to its neighborhood, which is the difference between the signal level at the bottom and the lower signal level of the two neighboring regions.

**Figure 5:**
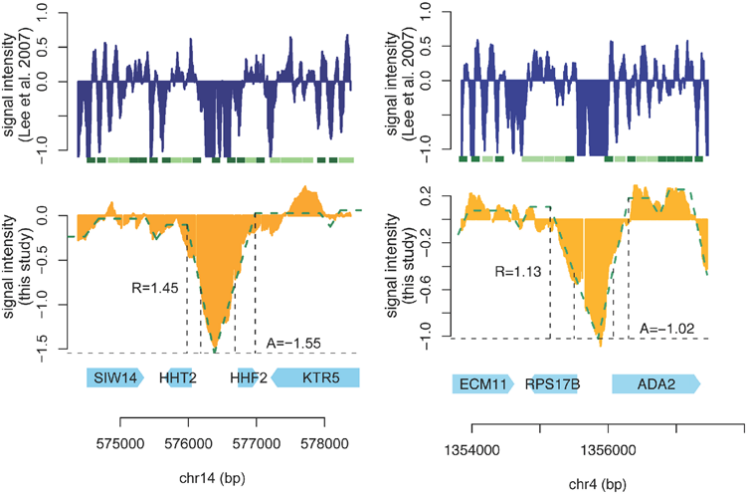
A comparison between the nucleosome occupancy data from this study and those from Lee et al. [6]. Two representative NFRs are shown using data from both this study and Lee et al. [6]. In the upper panels, trimmed raw nucleosome occupancy signals from Lee et al. are shown (blue), under which the rectangles show the nucleosome calls by HMM [5]. Dark and light green rectangles represent localized and delocalized nucleosomes respectively; white spaces represent linker regions. In the lower panels, the nucleosome binding is shown in yellow and the green broken lines are the SSMM output from this study. Vertical broken lines show the start, the end of the triangle/trapezoid patterns and the boundaries of NFRs. The absolute depletion (A) and relative depletion (R) are also annotated. The bottom panels show the names of ORFs in these regions. Similar figures for each of the 295 NFRs with highest DoND (*R*>1.0 and *A*<−1.0) can be downloaded at http://www.bios.unc.edu/~wsun/NFR/compare_Lee07_NFRs.pdf.

Applying SSMM to our nucleosome occupancy data led to the identification of 9593 NFRs in total, among which 35% are trapezoid patterns and 65% are triangle patterns. To determine the efficacy of SSMM in detecting the desired geometric shapes, we computed a goodness of fitness measure (*R* ^2^ value) for each detected NFR and found the value to be extremely high, hovering near 0.95 (see section 2.5 of in Supplementary Materials S1 for details). Figure 5 exemplifies how NFRs are identified by SSMM based on our tiling array data. The lower left panel shows an NFR located in the shared promoter region by genes HHT2 and HHF2 encoding histones H3 and H4 respectively. The lower right panel shows another NFR in the promoter region of RPS17B, encoding a ribosomal protein [21]. To compare SSMM outputs to those generated by HMM, we also show in Figure 5 the raw data and HMM calls from Lee et al. [6] in the upper panels. In contrast to SSMM, HMM outputs do not distinguish NFRs from the linker regions and do not provide DoND information for NFRs.

It is worth noting that despite the overall consistency, Lee et al.'s data generally depicts higher resolution nucleosome occupancy than our data, as a micrococcal nuclease based nucleosomal DNA isolation approach was used instead of a sonication based method employed in this study (see Methods). However, the micrococcal nuclease based protocol also introduces complexity in distinguishing NFRs from inter-nucleosomal linker regions. This is less problematic for our data as the linker regions are already smeared (see Figure 5). Our SSMM can also be applied to the micrococcal nuclease-based data after appropriate data smoothing.

**Figure 6:**
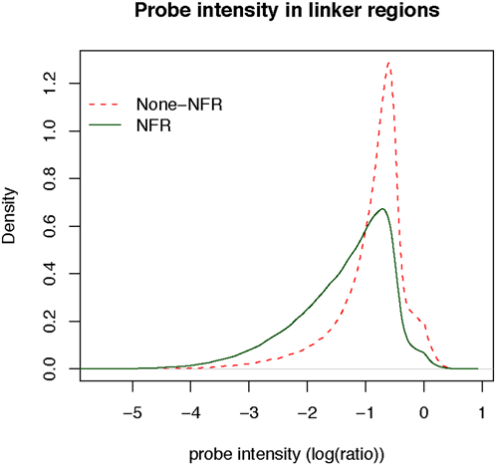
A comparison of intensities of linker probes between those that are inside NFRs and those that are outside of NFRs. The probe intensity is measured by the raw data of nucleosome occupancy, i.e., log(ratio) from Lee et al. [6]. A density distribution (green line) is plotted for the intensity of linker probes (defined in Lee et al. [6]) that are also present in NFRs (defined in this study). A similar curve was shown for linker probes that locate outside of NFRs (red line).

Both Yuan et al. [5] and Lee et al. [6] referred to all the regions that are not occupied by nucleosomes (well-positioned or delocalized) as the linker DNA. After taking a closer look at the probe intensity (the log ratio of nucleosome occupancy) within the linker DNA defined by Lee et al., we found that those genomic loci with lower probe intensities are more likely to fall into the NFRs that we identified (Figure 6). For example, among 314,457 linker probes with intensity higher than −1.0 (in log ratio), only 35.5% reside in NFRs, while for those 259,494 probes with signal lower than −1.0, 64% fall in NFRs. This agrees well with the anticipation that NFRs tend to have lower nucleosome occupancy than inter-nucleosomal linker regions.

### Effects of DoND on distributions and lengths of NFRs

The heterogeneity of nucleosome depletions at different chromosome features highlights the importance of quantifying the degree of nucleosome depletion. For each NFR, we introduced two measurements, absolute DoND (*A*) and relative DoND (*R*). These two measurements are well correlated as determined by plotting *R* against *A* for all 9593 NFRs detected in this work (Supplementary Figure 3 in Supplementary Materials S1). In fact, the relation between *A* and *R* can be approximated using a simple linear relation *A* = -*R*. Therefore to simplify the discussion, we shall use *R*>α and *A*<-α as the primary cutoff criteria for selecting NFRs according to DoND.

**Figure 7:**
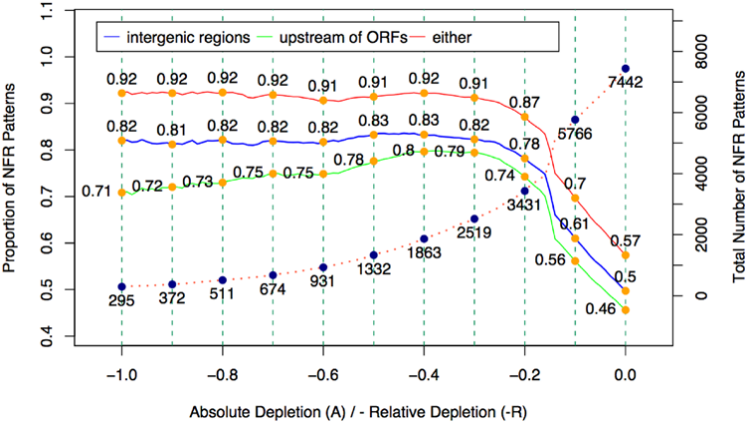
Mapping the locations of NFRs with various DoND. The proportions of NFR patterns located at intergenic regions, 500bp upstream regions of ORFs, and either regions given different cutoffs of DoND. Specifically, the cutoff α (α<0) indicates that the absolute depletion is smaller than α and the relative depletion is bigger than -α. The dash line indicates the total number of NFR patterns at different cutoffs (corresponding to the axis on the right side).

Nucleosomes are often depleted from promoters and intergenic regions [2], [3], [11]. Our results have also confirmed these observations. We further showed that DoND is a critical factor for the distribution of NFRs. NFRs with higher DoNDs are more likely to be located in the intergenic regions or upstream of coding regions (Figure 7, Supplementary Figure 4–5 in Supplementary Materials S1). In addition, those intergenic regions that are also upstream of coding regions are more likely to contain NFRs (Chi-square test p-value<5×10^−5^ for any DoND cutoffs from 0.2 to 1.0).

NFRs with a higher DoND are preferentially located in divergent intergenic regions, in which the neighboring genes share the 5′ upstream sequences. As the DoND increases, the proportion of NFRs located in divergent regions increases. In contrast the proportion in convergent regions (where the neighboring genes share the 3′ downstream sequences) decreases, and the proportion in tandem intergenic regions (where the neighboring genes are transcribed in the same direction) is roughly constant when the DoND increases (Supplementary Figure 6–8 in in Supplementary Materials S1).

Our data show that the occurrence of NFRs is related to DNA sequence properties, a relationship that is strengthened as DoND increases. We found the proportion of NFRs within TATA box-containing promoters [22] increases as the DoND increases (Supplementary Figure 9–11 in Supplementary Materials S1), which is consistent with a prediction by Segal et al. [11] using a computational model. Previous work has shown that TFBSs are over-represented in nucleosome-depleted promoters [2]. We further demonstrated that the proportion of NFRs harboring TFBSs increases as DoND increases (Supplementary Figure 12–14 in Supplementary Materials S1).

All previous studies identifying the locations and lengths of NFRs have been carried out only at the ensemble-level by averaging the nucleosome occupancy curves from a large number of promoter regions. Yuan et al. [5] reported a consensus NFR that is 150bp long at 200bp upstream of the ORF start site. Lee et al. [6] further identified a more coherent relation between the location of the consensus NFR and the TSS [23]. However, although the location and length of each individual NFR vary, neither paper provided such important information. In contrast, by characterizing individual NFRs using SSMM, we can study the location and length of each NFR in detail instead of merely conveying the “average” pattern.

**Figure 8:**
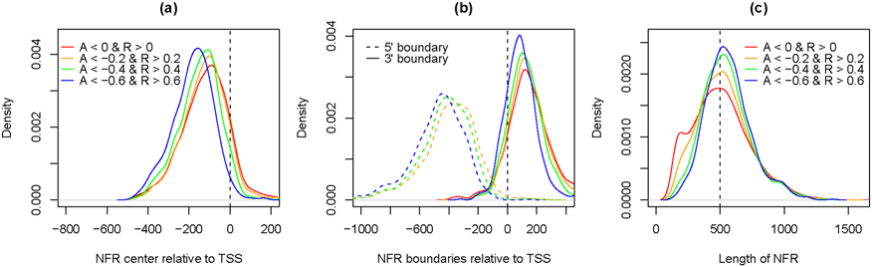
The distributions of locations and lengths of NFRs within promoter regions. NFRs that locate within 500bp upstream of ORFs were used and classified according to their DoND (A: absolute depletion, R: relative depletion). (a) The distributions of NFR positions (measured by the centers of NFR relative to TSSs) are plotted for NFRs with different DoND, as shown by curves with various colors. (b) The distributions of NFR boundaries (relative to TSSs) are plotted for NFRs with different DoND. The color codes are the same as in (a). (c) The distributions of NFR lengths are plotted for NFRs with different DoND.

We first examined the locations of NFRs relative to TSSs or ORFs. We found a total of 3448 NFRs at the promoter regions of 3447 distinct genes (within 500 upstream of ORFs), among which we obtained the TSS positions for 2601 ORFs [23]. Consistent with the aforementioned literature, the centers of these NFRs were found to lie around 100–200bp upstream of the TSS. Interestingly, NFRs with a higher DoND are observed further away from the TSS (i.e., shifted towards the 5′ direction) (Figure 8(a)). We found the starting point (5′) of NFRs appearing 350–450bp upstream of the TSS, and the end points (3′) of NFRs primarily occurring 100bp downstream of the TSS. The distributions of NFR boundaries (5′ and 3′) also shift to 5′ direction as DoND increases (Figure 8(b)). We obtained similar results (but with more variations) when measuring the positions of NFRs relative to the start codons of the ORFs (Supplementary Figure 15 in Supplementary Materials S1).

We next examined the distribution of NFR lengths. The majority of the NFRs within the promoter regions are found to be around 500bp, which is even more evident when the DoND increases (Figure 8(c)). A similar distribution was observed when using all NFRs (including those outside of the promoter regions) (Supplementary Figures 16–18 in Supplementary Materials S1). We conclude that NFRs have a typical length of 400–600bp, which is approximately the length of DNA wrapping around two-to-three nucleosomes plus linkers. Due to the variations in the locations and lengths of NFRs, most of them are expected to be longer than their consensus pattern derived by curve averaging (c.f. aforementioned results of [5], [6]).

### Factors of nucleosome depletion: transcriptional activity versus DNA affinity for histones

One of the long-standing puzzles in chromatin studies is the mechanism by which histones are evicted from NFRs. At least two major driving forces were reported. It has been shown that histones are depleted from active promoters by transcription-coupled machinery [2]–[5]. It is also known that certain DNA sequences have low intrinsic affinity for histones [7]–[11]. However, the relative effects of these two factors and their relationship remain unclear. We sought to address these questions based on the NFRs identified by our SSMM.

For each NFR, we first quantified the levels of transcriptional activity and DNA affinity for histones. To measure the transcriptional activity, a genome-wide RNA Polymerase II (Pol II) binding assay was performed using ChIP-chip (See Methods). We found that the DoND of NFRs correlates best with the Pol II binding levels within the neighboring regions of NFRs rather than those within the NFRs (Supplementary Tables 5–6 in Supplementary Materials S1). Presumably this is because NFRs typically occur at gene promoters, while the strongest Pol II binding mostly occurs on the neighboring coding regions, which could be either upstream or downstream of NFRs. Based on this observation, we averaged the Pol II binding signal within 1kb upstream or downstream of an NFR, and defined the higher signal as the local transcriptional activity. To measure the DNA affinity for histones within an NFR, we computed the average DNA affinity (measured as a probability of nucleosome occupancy) across all the nucleotides in that NFR using a previously published data set [11].

Next, in order to examine the contributions of transcriptional activity and DNA affinity in nucleosome depletion, we used a bivariate additive linear model with DoND (either absolute depletion or relative depletion) as a response and the two factors as covariates. The contribution of each factor is evaluated based on the variance of DoND it explains. It is worth noting that the linear regression and correlation analysis, as powerful tools to estimate the relative effect of different factors, however cannot be simply regarded as causal inference. Specifically, the variance of DoND is decomposed into three parts: the variance explained by DNA affinity, the variance explained by Pol II binding, and the covariance. We observed a positive covariance in all cases we considered and we referred to this covariance as the variance explained by both factors (See section 6 in Supplementary Materials S1 for details).

**Figure 9:**
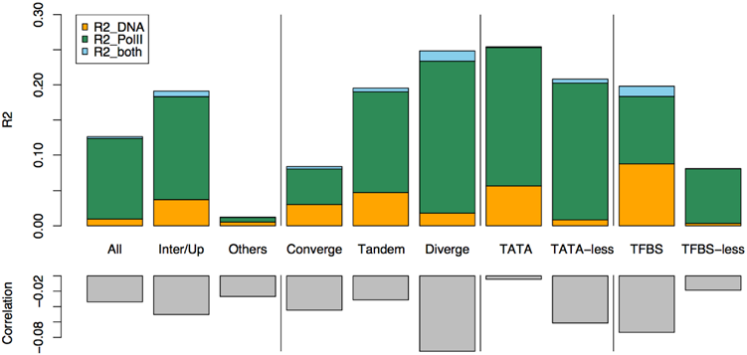
Dissection of the effects of DNA affinity for histones and Pol II for nucleosome depletion. The upper panel shows the total *R* ^2^ (percentage of variance) that can be explained by either DNA affinity for histones or transcriptional activity (Pol II binding), which is further divided into three parts: those explained by DNA affinity for histones (R2_DNA), by Pol II binding (R2_PolII), or by both (R2_both). Part of the variance can be explained by both factors due to their weak correlation between each other, as shown in the lower panel.

Initial examination of the entire 9593 NFRs by our additive bivariate linear model confirmed that both factors have significant effects on nucleosome depletion in NFRs, while the effect of transcriptional activity is dominant overall (Supplementary Table 6 in Supplementary Materials S1). For example, if we use absolute depletion to measure DoND, 12.6% of the DoND variance in total can be explained by either DNA affinity for histones or transcriptional activity, among which the majority (90.4%) is attributed to transcriptional activity. Only 7.6% is attributed to DNA affinity for histones, and less than 2% can be explained by either factor (Figure 9, see Supplementary Table 10 in Supplementary Materials S1 for details). Similar conclusions can be drawn using relative depletion to measure DoND (Supplementary Table 11 in Supplementary Materials S1).

To ask whether the effects of these two factors may differ in various NFR subgroups, we further compared the contribution of transcriptional activity and DNA affinity in the following NFR subgroups (Figure 9, see Supplementary Tables 6–11 in Supplementary Materials S1 for details of each subgroup): NFRs located in intergenic regions or 500bp upstream of ORFs (Inter/Up) or other genomic regions (Others); NFRs located in convergent (Converge), tandem (Tandem), and divergent (Diverge) intergenic regions; NFRs located in TATA-containing or TATA-less promoters (500bp upstream of ORFs); TFBS-containing NFRs or TFBS-less NFRs.

**Figure 10:**
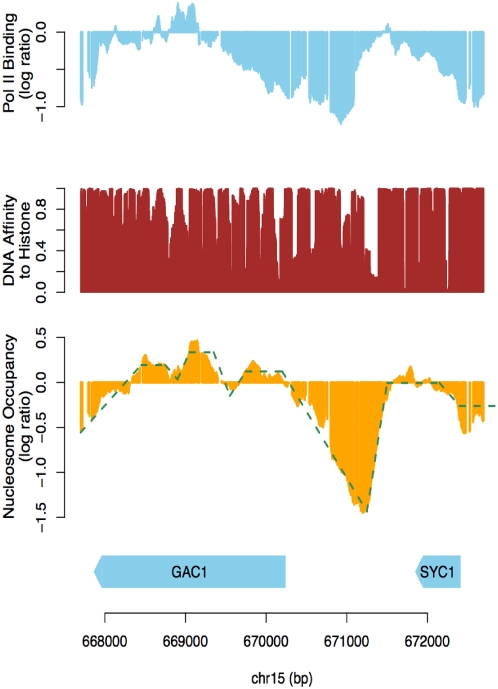
Histones are depleted from the promoter of gene *GAC1* prior to its activation. This figure shows the RNA polymerase II binding (log ratio from our ChIP-chip results), DNA affinity for histone (posterior probability of histone binding from Segal et al. [11]), and nucleosome occupancy (log ratio from our ChIP-chip results) around gene *GAC1* in rich medium.

Several interesting results were revealed. (1) While both Pol II and DNA affinity affect DoND in intergenic/promoter regions as expected, Pol II has an overall larger effect than DNA affinity, indicating that Pol II is a major deterministic factor of nucleosome depletion in intergenic/promoter regions. (2) Pol II binding explains more variance of DoND in regions with higher transcription activity, such as divergent and tandem intergenic regions. In contrast, DNA affinity has a larger effect in regions with less transcription activity such as convergent intergenic regions (Supplementary Tables 10–11 in Supplementary Materials S1). For example, when relative depletion is used as the measurement of DoND, DNA affinity contributes to 9%, 24.4%, and 78.7% of the total variance explained by the two factors in divergent, tandem, and convergent intergenic regions, respectively (Supplementary Table 11). (3) DNA affinity effect increases dramatically in NFRs containing TATA box or TFBS. For example, the contribution of DNA affinity increases 28 times more in TFBS-containing NFRs than in TFBS-less NFRs, and 6 times more in NFRs within TATA-containing promoters than TATA-less promoters (using absolute depletion, see Figure 9 and Supplementary Table 10 for details). In contrast, the Pol II binding effect remains comparable (less than 2 fold change) regardless of the presence of TFBS or TATA-box (Supplementary Table 10 in Supplementary Materials S1). Using relative depletion yields similar results (Supplementary Table 11 in Supplementary Materials S1). Interestingly, among those TFBS or TATA box-containing genes where DoND are dominated by DNA sequence properties, a number of them are stress response genes. For example, *GAC1* is a gene that is repressed in rich medium and induced upon diauxic transition when glucose is limited [24]. We found *GAC1* is depleted of nucleosomes at its promoter under a repressive state (Figure 10). *YMR279C*, a gene that is activated upon heat stress [25] and also loses histones from its promoter despite the fact that it is not transcribed (Supplementary Figure 19 in Supplementary Materials S1). Both promoter regions of these genes have been predicted to contain sequences that are poorly bound by nucleosomes [11]. Moreover, this may be a general phenomenon as it has been reported that TATA-box containing genes are highly enriched by stress-response genes [22]. The DNA sequences in the promoters of these genes could have evolved to enable strong repulsion against histones. In this way histones may be pre-cleared at these promoters prior to the entry of transcription machinery, presumably to allow the rapid binding of TBP (TATA-box binding protein) under environmental stresses. A similar mechanism could apply to TFBS-containing genes. Indeed, the low nucleosome occupancy on TATA-box has been reported to be encoded by the intrinsic properties of DNA sequences [11], [26]. The histone depletions around TFBSs were also observed elsewhere [2], [5], [6], [11]. However, a lingering question is that whether the depletion of histones in those TATA-box or TFBS containing promoters is simply due to their high transcriptional activity. Our study addressed this question by showing that low histone occupancy still exists even after excluding the transcription effect.

## Discussion

We have proposed an algorithm based on a structured segmental semi-Markov model, which extends hidden Markov model to detect and quantify segmental patterns using high resolution DNA-protein interaction data. In contrast to many algorithms designed to detect the binding of TFs, our algorithm is especially useful to characterize segmental features, which are commonly observed in epigenetic studies. We have applied this algorithm to a genome-wide nucleosome occupancy data and identified all NFRs across the entire yeast genome at 4bp high resolution. The location, length and DoND were quantified for each NFR. We showed that DoND, as measured by SSMM, closely associates with the distributions of NFRs.

We also studied the relative contributions of transcription machinery and DNA sequence in evicting histones from NFRs. The DoND measure we introduced plays a key role in formulating this biological question mathematically. A genome-wide RNA Polymerase II (Pol II) binding was used to evaluate the transcriptional activity based on ChIP-chip assay. We showed that Pol II and DNA play distinct roles in different types of NFRs. Taken together our study is a novel example of genome-wide investigations by combining transcription activity, genetic code and epigenetic information to address biological questions.

It is interesting to take a close look at the NFRs that are located outside of intergenic regions or the promoter regions. For example, with a stringent cutoff (*R*>0.4 and *A*<−0.4), we obtained 1863 NFRs with significant histone depletion. Although the majority of these NFRs are within intergenic regions or 500bp upstream of coding regions, we found there are still 145 NFRs (7.8%) located elsewhere (Supplementary Table 12 in Supplementary Materials S1). Among them, 52 are associated with tRNA genes, presumably due to their high transcription rate [22]. In addition there are 16 NFRs falling into ARS regions, which raises an interesting possibility for the involvement of NFRs in DNA replications. We also found 58 NFRs lie in 58 distinct ORFs (25 verified ORFs, see Supplementary Table 13 in Supplementary Materials S1; 12 uncharacterized and 21 dubious ORFs, see Supplementary Table 14 in Supplementary Materials S1). It is possible that these genic NFRs may harbor regulatory regions for the neighboring genes that are located more than 500bp away. Alternatively, these NFRs could affect genes in which they reside. For example, they may contain cryptic transcription start sites within coding regions allowing transcription to initiate there under certain conditions [26], [27]. Therefore our study highlights the need for further study towards the functional roles of NFRs within genic regions.

It is also worth noting that our methodology is not limited to the tiling array based NFR study as demonstrated above. The generality of our method allows for flexible applications to other high-throughput data in computational biology that may exhibit segmental patterns. For example, SSMM can be applied to the nucleosome occupancy data generated using micrococcal nuclease [6] or ChIP-seq [8] after appropriate smoothing. Moreover, our method also allows the choice of different segmental models depending on research interests, which make it general enough to be adapted for analyzing many other types of genomic data including those for DNA replication [28]–[30] and chromosome translocation [31]–[33].

## Materials and Methods

### ChIP-chip assay and data pre-processing

Three sets of ChIP-chip data were used in this study. The first set of ChIP-chip data of histone H3 was published previously [27]. Briefly, yeast chromatin was sheared by sonication into fragments with an average size of 500bp. Chromatin Immunoprecipitation (ChIP) was performed using antibody against histone H3 (a kind gift from Dr. Alain Verreault [28]), and then DNA crosslinked with nucleosomes was extracted and purified. Immunoprecipitated DNA was amplified and hybridized to Affymetrix Saccharomyces cerevisiae Tiling 1.0R Array to map the nucleosome occupancy along chromosomes in a 4-bp high-resolution manner. Raw intensities were computed by the Two-Sample Analysis method using Affymetrix Tiling Analysis Software v1.1. The tiling array features 2,635,714 oligo probes (25-mer) with 4 bp gaps (i.e. 21 bp overlaps) between the majority (91.5%) of adjacent probes. Only less than 1% of neighbor probes are separated by gaps longer than 20 bp. The entire yeast genome except centromeres is well represented on the arrays.

We further generated a new histone H3 ChIP-chip data set (two biological repeats) with a commercial H3 antibody (Abcam ab1791) using Affymetrix tiling arrays. The data obtained from these two antibodies are highly consistent (see Results). The average of all four repeats was used in our study.

An alternative approach to isolate nucleosomal DNA was employed in Yuan et al. [5] and Lee et al. [6], in which micrococcal nuclease (MN) was used to digest the linker DNA. While MN-based method produces relatively high resolution of nucleosome mapping, both approaches have been widely used and produce consistent results as shown in the paper.

In order to quantify the transcriptional activity at each genomic locus, we also measured genome-wide RNA Polymerase II (8WG16, Upstate) binding by ChIP-chip in a manner similar as for histone H3.

We have deposited related array data to ArrayExpress (http://www.ebi.ac.uk/microarray-as/ae/) with the accession number: E-MEXP-1951.

### Overview of SSMM

Segmental semi-Markov model (SSMM) is an extension from hidden Markov model (HMM). Compared to standard HMM, SSMM has two major generalizations. First, SSMM uses explicit state length density instead of the implicated exponential density [29]. For a standard HMM with transition probability *a_ii_* from state *s_i_* to itself, the probability that *d* consecutive observations are emitted from state *s_i_* is (*a_ii_*)*^d^* ^-1^(1-*a_ii_*). This probability decreases exponentially as *d* increases, which makes long segments impossible. For example, if *a_ii_* = 0.9 and there is one probe per 4bp, the probability that an NFR is longer than 500bp (typical length of NFR, see result section) is smaller than 2×10^−7^. Thus adaptation of explicit state length density is especially important for high density tilling array data. The drawback of explicit state length density is that there are more parameters to estimate, which may lead to over-fitting when sample size is small. However, for tilling array with millions of probes, over-fitting is unlikely to be a problem. Second, SSMM employs a segmental model to calculate the emission probability so that dependency is allowed for all observations within one segment [30]. This is desirable in analyzing tilling array data because this dependency assumption is more realistic. Furthermore, the segmental model can provide quantitative outputs characterizing the shapes of signal patterns. Both HMM and SSMM have been applied in speech recognition [29], [30]. HMM has been introduced to computational biology for sequence alignment and gene detection [31]–[33], as well as identifying TFBSs [13], [15] and NFRs [5]. However, despite its flexibility in handling high density data, SSMM has not been widely used for genomic studies. One possible reason is its heavy computational burden. The algorithm we introduced in this study incorporates several modifications of regular SSMM, which greatly improve the computational efficiency. We also designed the different hidden states and likelihood evaluation scheme to fit the purpose of NFR identification.

### Design of SSMM

Our SSMM is designed to capture two types of NFR patterns: triangle and trapezoid patterns. We organized the data into three hierarchical levels: probe, bin, and segment (Figure 3). We organized data into “bins” before “segments” for the following two reasons. First, it greatly reduces the computation burden by enforcing all the probes in one bin having only one underlying state. Second, discrete time SSMM assumes equally spaced observations. However, in our data, the gaps between adjacent probes are not constant. Grouping probes into bins ensures the distances between most adjacent bins are constant. Other possible solutions include modeling the transition probability between adjacent probes as a function of their distance [34], or implementing continuous time SSMM. However, these methods would significantly increase the algorithm complexity and computation time.

The bin size was empirically determined for the following considerations. On one hand, each bin should be long enough to include enough probes for linear model fitting. Long bins also help filter out noise and reduce computation burden. On the other hand, the signals will be over-smoothed if bins are too long. We use a bin of 50bp which on average covers 10–12 probes. This allows enough data points for model fitting while also avoids over-smoothing, given the lengths of NFRs typically vary from several hundred to a few thousand base pairs (see Result).

We compute the emission probability segment by segment. For one segment, we simply fit a linear model using nucleosome occupancy (log ratio) as the response and probe location as the covariate and then we calculated the emission probability based on the residuals. In order to obtain continuous prediction of nucleosome occupancy, we require the fitted line to start from the end point of previous segment. More details regarding the emission probability calculation are included in section 2.2 of Supplementary Materials S1.

Several parameters of SSMM need to be estimated: the transition probabilities from state 3 to state 2/4 (other transition probabilities are fixed as 0 or 1) and the probability distributions of state durations. These parameters are estimated by an iterative procedure as following. After generating the initial parameter values by uniform distributions, we first identify the most likely path (“best path”) by Viterbi algorithm [29]. We then estimate the parameters based on the most likely path, and iterate until the parameter estimations converge. The Viterbi algorithm for SSMM is a dynamic programming algorithm, which is similar to the one for HMM. The difference is that in order to determine the best path ended at time *k*, state *i*, in addition to choose the previous state *j*, we also need to choose the duration of state *i*. The details of Viterbi algorithms are discussed in section 2.3 of in Supplementary Materials S1. Given the “best path”, the parameters are estimated in the following way. We estimate the transition probabilities by the corresponding proportions of transitions. The duration probabilities are estimated by the proportions of observed durations. For example, in order to estimate *P*(duration of state *i* = *k*), we first take all the durations of state *i*, and then calculate the proportion of the durations of length *k*. For our data, it takes 7 iterations for the SSMM to converge; the convergence criterion is that the maximum change of either transition probabilities or duration probabilities is smaller than 10^−5^. One commonly used method for parameter estimation in HMM/SSMM is Baum-Welch algorithm (an EM algorithm) [29]. We do not use this EM algorithm for two reasons. First, as mentioned before, we wish to obtain continuous prediction of nucleosome occupancy; however this is not feasible using EM algorithm. Second, even we allow discontinuous prediction, the EM algorithm is computationally demanding, taking more than 30 times of CPU time compared to our iterative Viterbi algorithm (which takes about one day to finish all the computation). Further details about the algorithm and the parameter estimations are provided in section 2.2 and 2.4 of Supplementary Materials S1.

To investigate if our algorithm is easily trapped in a local optimum, we have also tested different initial parameter values. For example, we initiated the state transition/duration distributions from different normal distributions. We also initiated the lengths of states 1 and 2 from the empirical distributions of the lengths of chromosome features and intergenic regions respectively. In all cases we obtained almost identical final outputs. We have implemented our algorithm in an R package, ss.hmm, which can be downloaded at http://www.bios.unc.edu/~wsun/software.htm.

## Supporting Information

Supplementary Materials S1 Supplementary Methods and Results
(0.76 MB PDF)
