# Supplementary material for: Dissecting Nucleosome Free Regions by a Segmental Semi-Markov Model
Source: PLoS One. 2009 Mar 6;4(3):e4721. doi: 10.1371/journal.pone.0004721 (PMC2648986; doi:10.1371/journal.pone.0004721)
Supplement: Supplementary Materials S1 — Supplementary Methods and Results (0.76 MB PDF) [file pone.0004721.s001.pdf]

Supplementary Materials S1 for "Dissect nucleosome free regions by a segmental semi-Markov model"

Wei Sun*, Wei Xie*, Feng Xu, Michael Grunstein, Ker-Chau Li

February 4, 2009

* These authors contribute equally to this work

Abbreviations:

DoND, degree of nucleosome depletion;

HMM, hidden Markov model;

SSMM, segmental semi-Markov model;

TFBS, transcription factor binding site;

NOR, nucleosome occupied region;

NFR, nucleosome free region.

Contents

1 Compare TF binding signal and NFR signal **3**

2 Algorithm of Segmental Semi-Markov Model **3**

2.1 Notations 3

2.2 Segmental model fitting and emission probability calculation 5

2.3 Algorithms 6

2.3.1 Viterbi 7

2.3.2 Forward 9

2.3.3 Backward 10

2.3.4 Posterior probability 11

2.4 Parameter estimation 12

2.5 R^2 estimation 14

3 Analysis of the raw data of nucleosome occupancy **16**

3.1 Data validation 16

3.2 Nucleosome occupancy at various chromosomal features 18

4 Compare absolute depletion and relative depletion of NFRs **20**

5 Distributions and lengths of NFRs with different DoND **21**

6 Nucleosome depletion forces: DNA affinity for histones and transcriptional activity **35**

7 NFRs outside of the promoters or intergenic regions **44**

1 Compare TF binding signal and NFR signal

This figure shows the comparison of the signal patterns between a TF binding and an NFR (Nucleosome Free Region) from tilling DNA microarray data.

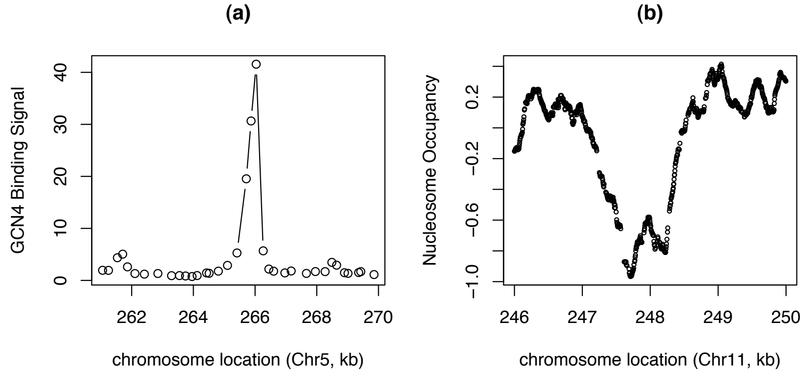

Supplementary Figure 1: A comparison of TF binding signal and nucleosome occupancy signal. The TF (Gcn4) binding data were reported previously[1]. Similar pattern can be observed elsewhere [2]. The nucleosome occupancy data (H3) is obtained in this study, which has higher resolution.

2 Algorithm of Segmental Semi-Markov Model

2.1 Notations

Segmental Semi-Markov Model (SSMM) has been used in speech recognition [3, 4]. We modified the conventional SSMM algorithm mainly in two aspects:

1. We organized the input data in a hierarchical structure: probe $\rightarrow$ bin $\rightarrow$ segment, so that we do not require the time points to be strictly evenly spaced.

2. We required the continuity of consecutive segmental models.

We denote the parameters of a segmental semi-Markov model as $\Lambda = \{\pi, A, L, D, d_i(\cdot), e(\cdot)\}$), which include 6 components:

1. π: the initial probability, $\pi(i) = P(q_1 = i)$, where q_t indicates the state at time t, and i indicates the ith state, $1 \leqslant i \leqslant I$, I is the total number of states.

2. $A = \{a_{ij}\}$: the transition probability, $a_{ij} = P(q_{t+1} = j | q_t = i)$.

3. L: length of each bin.

4. D: the maximum duration.

5. $d_i(\cdot)$: the density of duration of state i.

6. $e(\cdot)$: emission probability.

Suppose there are n observations (i.e., n probes in this NFR study). We denote the observations as $X = \{x_1, x_2, ..., x_n\}$ and the corresponding time points (or probe locations) as $T = \{t_1, t_2, ..., t_n\}$. Thus the total number of bins, denoted by Z, is $Z = \texttt{ceil}(n/L)$. We use $\{t_{s_1}, ..., t_{s_Z}\}$ to indicate the start of each bin and use $\{t_{e_1}, ..., t_{e_Z}\}$ to indicate the end of each bin. We use $\texttt{seg}(i, p, q)$ to indicate a segment from bin p to bin q (including p and q) with underlying state i. Without requirement of continuity, previous works of SSMM defined the emission probabilities of one segment $\texttt{seg}(i, p, q)$ as $e_i(p, q) = P(x_{s_p}, x_{s_p+1}, ..., x_{e_q} | s(p, q) = i)$, where $s(p, q)$ are states from bin p to bin q. In this study, we use linear model as segmental model, and require the linear model for segment $\texttt{seg}(i, p, q)$ pass the predicted end point of previous segment $\texttt{seg}(j, o, p-1)$. Thus we define the emission probability based on the previous

segment as well: $e_{ji}(o,p,q) = P(x_{s_p}, x_{s_p+1}, ..., x_{e_q} | s(p,q) = i, s(o,p-1) = j)$, where $1 \leqslant o \leqslant p-1$.

2.2 Segmental model fitting and emission probability calculation

Our SSMM model for NFR detection is illustrated in the main text. We use a linear model as the segmental model and we list below additional details of segmental model fitting and emission probability calculation specifically designed for NFR detection.

1. Given the end point of previous segment, the linear model for states 1 and 2, which are horizontal lines, are already decided. Thus there is no need for model fitting.

2. Given the end point of previous segment, denoted by $(t_{\texttt{prev}}, x_{\texttt{prev}})$, the linear model for states 3 and 4 is $(x_w - x_{\texttt{prev}}) = b(t_w - t_{\texttt{prev}})$, where w is index of those observation in the current segment of state 3 or 4. The coefficients b can be estimated by least square method. Specifically,

$$b = \sum_w (x_w - x_{\texttt{prev}})(t_w - t_{\texttt{prev}}) / \sum_w (t_w - t_{\texttt{prev}})^2.$$

3. We denote the residuals as $r_w = x_w - \hat{x}_w$, where $\hat{x}_w$ is fitted value from the segmental (linear) model. The emission probability (likelihood) of the segment is calculated by assuming the residuals are from normal distribution with mean 0 and a maximum likelihood estimation of the variance $\hat{\sigma}^2 = \sum_w r_w^2 / n_{\texttt{seg}}$, where $n_{\texttt{seg}}$ is the number of observations in the segment.

4. The segmental models of states 3 and 4 have one more parameter than state 1 or 2, the slope. Penalized likelihood should be calculated, e.g., AIC or BIC. In this study, we used BIC.

5. The triangle/trapezoid patterns with very small slopes on the two edges can be frequently caused by data noise and are not of interest to us. They may also cause over-fitting. Thus if the absolute value of an estimated slope is smaller than 0.001, we forced it to be -0.001 or 0.001 in order to calculate the emission probabilities for state 3 or 4 respectively.

2.3 Algorithms

Analogous to the algorithms in regular HMM, we presented the following four algorithms for SSMM: Viterbi, forward, backward, and posterior probability. The "Viterbi" algorithm finds the most likely complete path, while the forward and backward algorithm together identify the posterior probability of which state one bin is emitted from.

Viterbi algorithm is favored in our model fitting for the following reasons. As one of the challenges in our model fitting, estimation of the emission probability $e_{ji}(o, p, q)$ between bin p and q requires the knowledge of the end point of the previous segmental model from bin o to $p-1$. This can be easily obtained in "Viterbi" algorithm since when we calculate the emission probability of one segment, the most likely path in the previous segments are already known. However, in forward and backward algorithm, we only know that the previous segment is from bin o to $p-1$, corresponding to state j, i.e., $\texttt{seg}(j, o, p-1)$. The calculation of the end point of $\texttt{seg}(j, o, p-1)$ requires knowledge of where the segment before bin o ends, which is unknown. This difficulty

can be bypassed if we do not require the continuity of the fitted curve. The starting point of $\texttt{seg}(j, o, p-1)$ can be set to be free allowing calculation of its end point, which can be used as the start point of the segment $\texttt{seg}(i, p, q)$. However, another limitation of the forward-backward algorithm is that it takes much more computation time than Viterbi algorithm, which is critical for high resolution tiling array data analysis. For instance, the Viterbi algorithm is 34 times faster than forward-backward algorithm for the model fitting of a 3000-probe segment (on a 2GHz Intel Core Duo MacBook Pro, 1GB RAM). Given that it takes around 1 day for the Viterbi algorithm to finish all the model fitting and parameter estimations for the entire genome, approximately one month is needed for forward-backward algorithm.

The following algorithms are implemented in a R package ss.hmm, which can be freely downloaded at http://www.bios.unc.edu/∼wsun/software.htm. In order to avoid underflow, we carried out all the calculations in log scale. A function $\texttt{logsumexp}(v)$ is used during the calculation:

$$\texttt{logsumexp}(v) = \log\left(\sum_{g=1}^{G} \exp(v_g)\right) \tag{1}$$

where $v = \{v_1, v_2, ..., v_G\}$ is a vector.

2.3.1 Viterbi

Input

$X = \{x_1, x_2, ..., x_n\}$, $T = \{t_1, t_2, ..., t_n\}$ and parameters $\Lambda = \{\pi, A, L, D, d_i(\cdot), e(\cdot)\}$, where X are observations and T are the corresponding time.

Ouput

$\texttt{path}(t_w)$: $1 \leq w \leq n$, the most probable path along time T.

Intermediate Variables

$\texttt{p}(k,i)$: the maximum probability that state i ends at bin k, $\texttt{log.p}(k,i) = \log(\texttt{p}(k,i))$.

$\texttt{dura}(k,i)$: the duration the segment that is emitted from state i and ends at bin k.

$\texttt{prev}(k,i)$: the state before the segment that is emitted from state i and ends at bin k.

Algorithm

1. Calculate Intermediate Variables

   For the first bin, $k = 1$,

$$\begin{aligned} \texttt{p}(1,i) &= \pi_i d_i(1) e_i(1,1) && (2) \\ \texttt{log.p}(1,i) &= \log(\pi_i) + \log(d_i(1)) + \log(e_i(1,1)) && (3) \\ \texttt{dura}(1,i) &= 1 && (4) \end{aligned}$$

   For $k \geqslant 2$, suppose the previous state is j. We use d to indicate the duration of state i, $1 \leqslant d \leqslant \min(k-1, D)$. The start point of previous segment is $k' = k - d - \texttt{dura}(k-d, j) + 1$.

$$\begin{aligned} p(k,i,d,j) &= \texttt{p}(k-d,j) a_{ji} d_i(d) e_{ji}(k', k-d+1, k) && (5) \\ \log(p(k,i,d,j)) &= \log(\texttt{p}(k-d,j)) + \log(a_{ji}) + \log(d_i(d)) \\ &\quad + \log(e_{ji}(k', k-d+1, k)) && (6) \end{aligned}$$

   If $k \leqslant D$, it is possible that state i begins from the first time point, then

$$\begin{aligned} p(k,i,d=k,j=NULL) &= \pi_i d_i(k) e_i(1,k) && (7) \\ \log(p(t,i,d=k,j=NULL)) &= \log(\pi_i) + \log(d_i(k)) + \log(e_i(1,k)) && (8) \end{aligned}$$

Then we can calculate the best path ended at time k, state i by

$$\begin{aligned}
\texttt{p}(k,i) &= \max_{d,j} p(k,i,d,j) && (9)\\
\texttt{log.p}(k,i) &= \max_{d,j} \log(p(k,i,d,j)) && (10)\\
\texttt{dura}(k,i) &= \texttt{argmax}_d \log(p(k,i,d,j)) && (11)\\
\texttt{prev}(k,i) &= \texttt{argmax}_j \log(p(k,i,d,j)) && (12)
\end{aligned}$$

2. Trace back the best path

$$\texttt{path}(Z) = \texttt{argmax}_i(\texttt{log.p}(Z,i)) \quad (13)$$

then find the previous segment that corresponds to state $\texttt{prev}(Z, \texttt{path}(Z))$ and ends at time $Z - \texttt{dura}(Z, \texttt{path}(Z))$. Keep recurring to find the entire path.

2.3.2 Forward

Input

$X = \{x_1, x_2, ..., x_n\}$, $T = \{t_1, t_2, ..., t_n\}$ and parameters $\Lambda = \{\pi, A, L, D, d_i(\cdot), e(\cdot)\}$.

Ouput

The forward probabilities for state i from bin p to q: $f(i,p,q) = P(x_1, ..., x_{e_q}, q(p,q) = i|\Lambda)$, where $1 \leqslant p \leqslant q \leqslant Z$

Algorithm

Initialization

$p = 1,\ q = \{1, ..., \min(Z, D)\}$:

$$\begin{aligned}
f(i,1,q) &= \pi(i) d_i(q) e_i(1,q) && (14)\\
\log(f(i,1,q)) &= \log(\pi(i)) + \log(d_i(q)) + \log(e_i(1,q)) && (15)
\end{aligned}$$

Recursion

$p = \{2, ..., Z\}$, and for each p, $q = \{p, ..., \min(p + D - 1, Z)\}$, $o = \{\max(1, p - D), ..., p - 1\}$.

$$
\begin{aligned}
f(i,p,q) &= \sum_{j \neq i} \left[\left[\sum_{o} f(j,o,p-1) e_{ji}(o,p,q) \right] a_{ji} \right] d_i(q-p+1) \quad (16) \\
\log(f(i,p,q)) &= \texttt{logsumexp}_{j \neq i} [\texttt{logsumexp}_o [\log(f(j,o,p-1)) + \log(e_{ji}(o,p,q))] \\
&\quad + \log(a_{ji})] + \log(d_i(q-p+1)) \quad (17)
\end{aligned}
$$

2.3.3 Backward

Input

$X = \{x_1, x_2, ..., x_n\}$, $T = \{t_1, t_2, ..., t_n\}$ and parameters $\Lambda = \{\pi, A, L, D, d_i(\cdot), e(\cdot)\}$

Ouput

The backward probabilities for state i from bin p to q: $b(i,p,q) = P(x_{s_q+1}, ..., x_n | q(p,q) = i, \Lambda)$, where $1 \leqslant p \leqslant q \leqslant Z$

Algorithm

Initialization

$$
\begin{aligned}
b(i,p,Z) &= 1 \quad (18) \\
\log(b(i,p,Z)) &= 0 \quad (19)
\end{aligned}
$$

Recursion

$q = \{Z-1, ..., 1\}$, and for each q, $p = \{\max(1, q-D+1), ..., q\}$, $r = \{q+1, ..., \min(q+$

$D, Z)\}$.

$$b(i,p,q) = \sum_{j\neq i}\left[a_{ij}\left[\sum_r e_{ij}(p,q+1,r)d_j(r-q)b(j,q+1,r)\right]\right] \quad (20)$$

$$\log(b(i,p,q)) = \texttt{logsumexp}_{j\neq i}[\log(a_{ij}) + \texttt{logsumexp}_r[\log(e_{ij}(p,q+1,r)) + \log(d_j(r-q)) + \log(b(j,q+1,r))]] \quad (21)$$

2.3.4 Posterior probability

Calculate the posterior probability based on forward and backward algorithm.

Input

forward probability $\{f(i,u,v)\}$ and backward probability $\{b(i,u,v)\}$, where i ($1 \leqslant i \leqslant I$) indicates the state and u and v ($1 \leqslant u \leqslant v \leqslant Z$) indicate the bins.

Ouput

$p_i(k)$: posterior probability $P(q(k) = i|X, \Lambda)$, where $q(k)$ indicates state of the k-th bin.

Algorithm

$$\begin{aligned} p_i(k) &= P(q(k)=i|X,\Lambda) = \frac{P(q(k)=i, X|\Lambda)}{P(X|\Lambda)} \\ &\sim P(q(k)=i, X|\Lambda) \\ &= \sum_u \sum_v f(i,u,v)b(i,u,v) \end{aligned} \quad (22)$$

where $\max(t-D+1, 1) \leqslant u \leqslant k$ and $k \leqslant v \leqslant \min(u+D-1, Z)$.

$$\log(p_i(k)) = C + \log(P(q(k)=i|X,\Lambda)$$

$$= \quad C + \texttt{logsumexp}_u[\texttt{logsumexp}_v[\log(f(i,u,v)) + \log(b(i,u,v))]] \quad (23)$$

where C is a normalizatioin constant so that $\sum_i p_i(k) = 1$.

2.4 Parameter estimation

We need to estimate the transition probabilities from state 3 to state 2/4 (other transition probabilities are fixed as 0 or 1), and the probability distributions of state durations (See main text Figure 2 for description of the states). For HMM, parameters are usually estimated by Baum-Welch algorithm (an EM algorithm) [3, 5]. However, as explained in previous section, this EM algorithm cannot be applied to our SSMM because we require the continuity of the fitted curve. Even if we do not require the continuity of the fitted curve, the EM algorithm that used forward-backward algorithm takes much more time than Viterbi algorithm. Therefore we choose to use Viterbi algorithm [3, 5] for parameter estimation. With one set of initial parameters, we can generate the most likely path, which is used to update the parameter estimations, and iterate until the parameter estimations converge. The most likely path at convergence is our final result.

The difference between the algorithm we used and Baum-Welch algorithm is analogous to the difference between (hard) K-mean clustering and soft K-mean (EM) clustering. For hard K-mean, one point is assigned to the most likely cluster, while for soft K-mean, posterior probabilities of cluster memberships are estimated. Similarly, in our SSMM algorithm, we assume one bin is emitted from the most likely state, while in Baum-Welch algorithm, posterior probabilities of underlying states are used.

We do not wish to make restrictions on the distribution functions of duration or tran-

sition probabilities. We start with the uniform distributions. The initial transition matrix is:

$$\begin{pmatrix} 0 & 0 & 1 & 0 \\ 0 & 0 & 0 & 1 \\ 0 & 0.5 & 0 & 0.5 \\ 1 & 0 & 0 & 0 \end{pmatrix}$$

where the number in i-th row and j-th column is the transition probability from state i to j: a_{ij}. The duration of each state is counted by the number of "bins" (each "bin" covers 50bp). The initial distributions of durations for state 1 to 4 are uniform(6,100), uniform(3,30), uniform(3,50), and uniform(3,50) respectively. The only restriction here is the ranges. We do not allow too short durations in order to avoid over-fitting. The maximums of durations are set to be large enough to cover all possible durations. At convergence, the transition probabilities are $a_{32} = 0.37$, $a_{34} = 0.63$. The following Supplementary Figure 2 shows the distribution of state durations at convergence of parameter estimation, where X-axis is the duration in base pair, and Y-axis is the frequency.

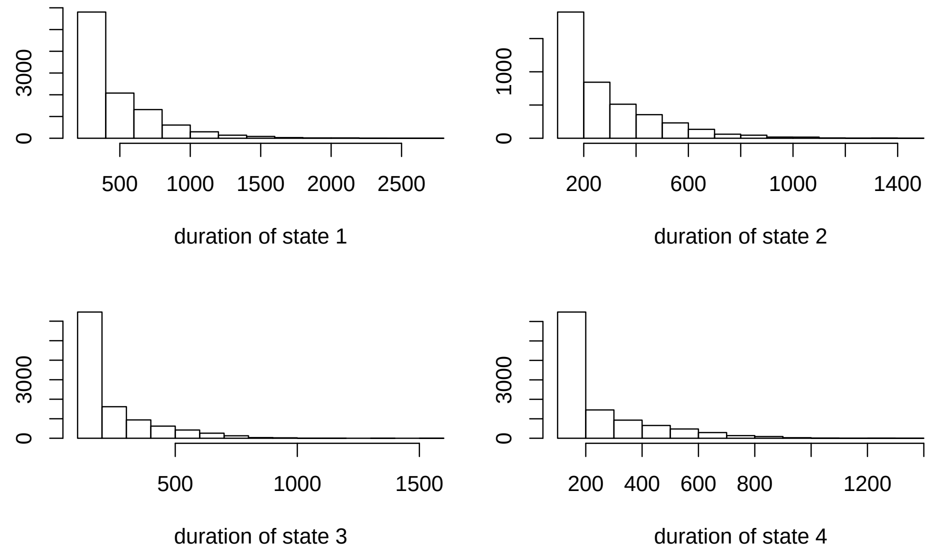

Supplementary Figure 2: Distribution of state durations after convergence

2.5 R^2 estimation

After fitting the SSMM, we can obtain the predicted intensity for each probe. Denote the observed and data at probe i and y_i, the predicted value as $\hat{y}_i$, and the residual as r_i, that is

$$y_i = \hat{y}_i + r_i \tag{24}$$

We use n to denote the sample size and use $\bar{y}$ to denote the sample mean of y, then the sample variance of y can be decomposed as

$$\begin{aligned} \text{var}(y) &= \frac{1}{n-1}\sum_{i=1}^{n}(y_i - \bar{y})^2 & (25) \\ &= \frac{1}{n-1}\left[\sum_{i=1}^{n}(y_i - \hat{y}_i)^2 + \sum_{i=1}^{n}(y_i - \hat{y}_i)(\hat{y}_i - \bar{y}) + \sum_{i=1}^{n}(\hat{y}_i - \bar{y})^2\right] & (26) \end{aligned}$$

In the situation of linear model least-sqaure fitting, the term $\frac{1}{n-1}\sum_{i=1}^{n}(y_i-\hat{y}_i)(\hat{y}_i-\bar{y})$ is 0, therefore the sample variance of y can be decomposed as the summation of the residual variance $\frac{1}{n-1}\sum_{i=1}^{n}(y_i-\hat{y}_i)^2$ and the variance explained by the model fitting $\frac{1}{n-1}\sum_{i=1}^{n}(\hat{y}_i-\bar{y})^2$, and R^2 is defined as

$$R^2 = \frac{\sum_{i=1}^{n}(\hat{y}_i-\bar{y})^2}{\sum_{i=1}^{n}(y_i-\bar{y}_i)^2} = 1 - \frac{\sum_{i=1}^{n}(y_i-\hat{y}_i)^2}{\sum_{i=1}^{n}(y_i-\bar{y}_i)^2} \tag{27}$$

By our SSMM model fitting, the term $\frac{1}{n-1}\sum_{i=1}^{n}(y_i-\hat{y}_i)(\hat{y}_i-\bar{y})$ may not be 0 because we have restrictions regarding the start point of each segment in order to obtain a continuous curve across the genome. Nevertheless, as long as the linear model is a resonable segmental model, the term $\frac{1}{n-1}\sum_{i=1}^{n}(y_i-\hat{y}_i)(\hat{y}_i-\bar{y})$ should be close to 0, and we can define a conservative estimation of R^2 as

$$R^2 = \min\left(\frac{\sum_{i=1}^{n}(\hat{y}_i-\bar{y})^2}{\sum_{i=1}^{n}(y_i-\bar{y}_i)^2}, 1 - \frac{\sum_{i=1}^{n}(y_i-\hat{y}_i)^2}{\sum_{i=1}^{n}(y_i-\bar{y}_i)^2}\right) \tag{28}$$

For example, by applying our SSMM to our nucleosome occupation data, we identified 9593 NFRs that cover 1,293,610 probes in total. Based on the result of our SSMM, we can obtain $\hat{y}_i \quad i = 1, ..., 1,293,610$ for each of these probes and the following numerical resutls:

$$\frac{1}{n-1}\sum_{i=1}^{n}(y_i-\bar{y}_i)^2 = 0.120758 \tag{29}$$

$$\frac{1}{n-1}\sum_{i=1}^{n}(y_i-\hat{y}_i)^2 = 0.006175 \tag{30}$$

$$\frac{1}{n-1}\sum_{i=1}^{n}(\hat{y}_i-\bar{y})^2 = 0.116389 \tag{31}$$

$$\frac{1}{n-1}\sum_{i=1}^{n}(y_i-\hat{y}_i)(\hat{y}_i-\bar{y}) = -0.000903 \tag{32}$$

Therefore the conservative estimation of R^2 is

$$R^2 = \min(0.116389/0.120758, 1 - 0.006175/0.120758) = 0.949 \tag{33}$$

3 Analysis of the raw data of nucleosome occupancy

3.1 Data validation

First we compared our nucleosome occupancy data with previously published genome-wide data in lower resolutions. Because our data have a higher resolution, we calculate Pearson's correlation using a coarse-grained version of our data. Supplementary Tables 1-3 list the correlations between our data and data from Bernstein et al. [6], Lee et al. [7], and Pokholok et al. [1] respectively.

Supplementary Table 1: Correlations between our data and the data by Bernstein et al.

Bernstein et al. [6] studied the occupancy of H2B and H3 in $\sim$ 6000 intergenic/promoter regions in yeast genome. For a coarse-grained version of our data we calculated either mean or median of our data in each of the 6000 intergenic/promoter regions used by Bernstein et al. [6].

	H3	H2B	Average_H3_H2B
Mean	0.68	0.56	0.66
Median	0.67	0.56	0.66

Supplementary Table 2: Correlations between our data and the data by Lee et al.

Lee et al. [7] examined the occupancy of H3 and H4 in $\sim$ 12000 intergenic regions and ORFs. For a coarse-grained version of our data we calculated either mean or median of our data in each of the 12000 intergenic regions used by Lee et al. [7].

	MycH4	H3	Average_MycH4_H3
Mean	0.75	0.71	0.78
Median	0.74	0.70	0.77

Supplementary Table 3: Correlations between our data and the data by Pokholok et al.

Pokholok et al. [1] performed ChIP-chip experiments for H3 and H4 using 60-mer Agilent DNA microarrays, which have $\sim$ 41000 probes covering 85% of the yeast genome. For a coarse-grained version of our data we calculated either mean or median of our data in each of the 41000 regions covered by probes used by Lee et al. [7].

	H4	H3	Average_H4_H3
Mean	0.68	0.48	0.62
Median	0.68	0.48	0.62

3.2 Nucleosome occupancy at various chromosomal features

As a preliminary investigation of regions with low nucleosome occupancy in yeast genome, we compared the nucleosome occupancies in various chromosomal features with those in intergenic regions (Supplementary Table 4). Chromosome features are defined by SGD [8], which include ORF (Open Reading Frame), ARS (Autonomously Replicating Sequence), rRNA, tRNA, snRNA (small nuclear RNA), snoRNA (small nucleolar RNA), rRNA, long terminal repeat, telomeric elements, introns and transposons. The comparison between chromosome features and intergenic regions was carried out by student t-test. One technical problem is that t-test is biased by the high dependency between signals of adjacent probes due to probe overlaps. Thus for each feature, we randomly selected one probe from each instance of the feature and one probe from each intergenic regions, forming two groups for t-test. This was repeated for 50 times and the medians and two quantiles of the t-statistics are reported in Supplementary Table 4. We found that while the majority of chromosomal features have higher nucleosome occupancies than intergenic regions, a few classes including tRNA, intron, cetromere, and snoRNA, however, have even lower nucleosome occupancies than intergenic regions.

Supplementary Table 4: Nucleosome occupancies at chromosome features vs. intergenic regions

In this table, $\mathbf{n}$ is the total number of instances of one chromosome feature. $\mathbf{n_{median}}$ is the median number of probes selected. In different permutations, the number of probes selected may be different because different instances of one feature may overlaps, thus $\mathbf{n_{median}}$ may be smaller than $\mathbf{n}$. $\mathbf{t_{median}}$ is the median of t-statistics. $\mathbf{p_{median}}$, $\mathbf{p_{25\%}}$, and $\mathbf{p_{75\%}}$ are median, 1st quantile, and 3rd quantile of the t-test p-values respectively. The features are ordered by medians of t-statistics.

**Feature**	**n**	$\mathbf{n_{median}}$	$\mathbf{t_{median}}$	$\mathbf{p_{median}}$	$\mathbf{p_{25\%}}$	$\mathbf{p_{75\%}}$
tRNA	299	275	-43.63	1.9e-147	1.9e-149	7.5e-146
snoRNA	75	75	-5.09	2.5e-06	1.4e-06	4.9e-06
intron	367	334	-2.66	0.0081	0.0051	0.018
ARS	248	248	-1.52	0.13	0.084	0.19
ncRNA	9	8	-1.35	0.22	0.18	0.27
telomeric repeat	31	31	-0.65	0.52	0.35	0.79
snRNA	6	6	-0.34	0.75	0.49	0.83
X element combinatorial repeats	28	28	5.45	8.4e-06	2.8e-07	4.3e-05
ARS consensus sequence	66	32	6.14	7.7e-07	4.8e-07	1e-06
telomere	32	32	8.84	4.2e-10	1.2e-11	3.6e-09
pseudogene	21	21	10.22	1.8e-09	5.8e-10	7.7e-09
X element core sequence	32	32	10.97	1.7e-12	2.3e-14	2.6e-11
Y' element	19	19	12.62	1.3e-10	3.2e-13	3.6e-08
rRNA	27	25	13.02	8.6e-13	1.7e-14	4.6e-11
retrotransposon	50	50	20.06	1.3e-26	1.6e-29	1.2e-24
transposable element gene	89	89	24.14	2.3e-44	1.2e-47	3.7e-41
ORF	6604	6574	49.97	0	0	0

4 Compare absolute depletion and relative depletion of NFRs

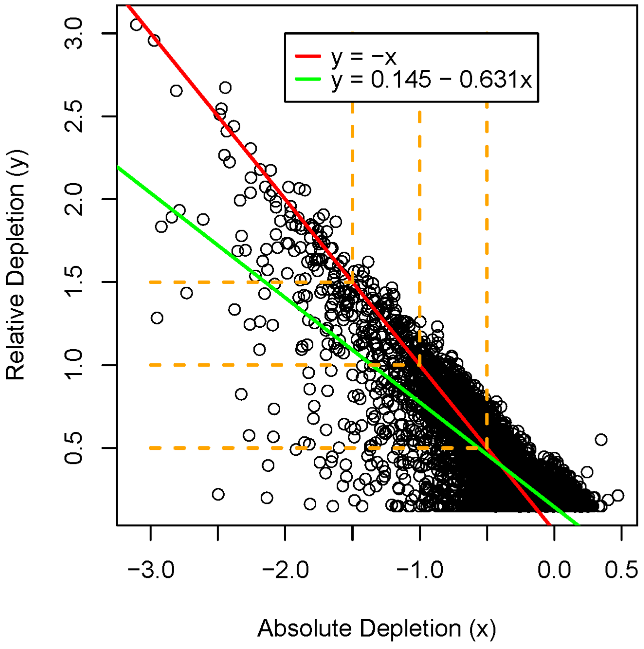

Supplementary Figure 3: Absolute depletion vs. relative depletion
R denotes relative depletion and A denotes absolute depletion. The red line indicates $R = -A$.

This scatter plot shows that the two measurements of nucleosome depletion are well correlated. We shall use $R = -A > \alpha$ as the primary cutoff criteria for selecting NFRs for further investigation.

5 Distributions and lengths of NFRs with different DoND

We systematically examined the proportions of NFRs in intergenic or the promoter regions (defined as 500bp upstream of coding regions). Information of intergenic regions and ORF start positions were downloaded from SGD [8]. The total length of intergenic regions is about 2.88Mb, or 23.9% of the 12.07Mb yeast genome. The 500bp upstream regions of 6604 ORFs occupy roughly 2.82Mb DNA sequences, or 23.3% of the yeast genome. About 1.83 Mb (15.2%) DNA sequence is both at intergenic regions and 500bp upstream of coding regions. As expected, NFRs with higher DoND are more likely located in intergenic or the promoter regions (Supplementary Figure 4-5). The enrichment of NFRs in intergenic regions or the promoter regions is highly significant (Chi-square test p-value $< 1e^{-80}$ for any cutoffs of absolute depletion and/or relative depletion from 0.2 to 1.0). In addition, those intergenic regions that are also upstream of coding regions are more likely to contain NFRs (Chi-square test p-value $< 5e^{-10}$ for any cutoffs from 0.2 to 1.0).

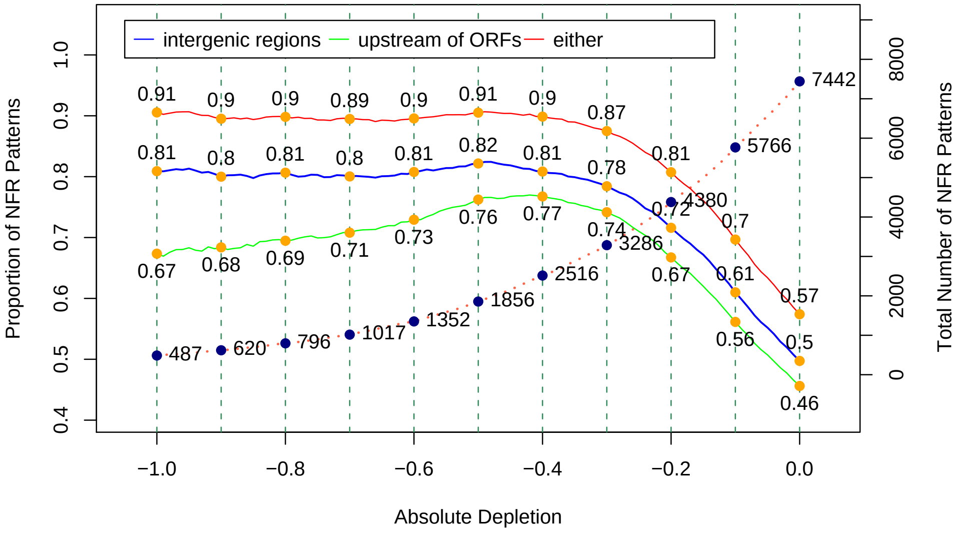

Supplementary Figure 4: Locations of NFRs vs. absolute depletion

The proportions of NFR patterns located in intergenic regions, 500bp upstream of ORFs, and either intergenic or 500bp upstream region according to different cutoffs of absolute depletion. The dash line indicates the total number of NFR patterns at different cutoffs of absolute depletion (corresponding to the axis on the right side).

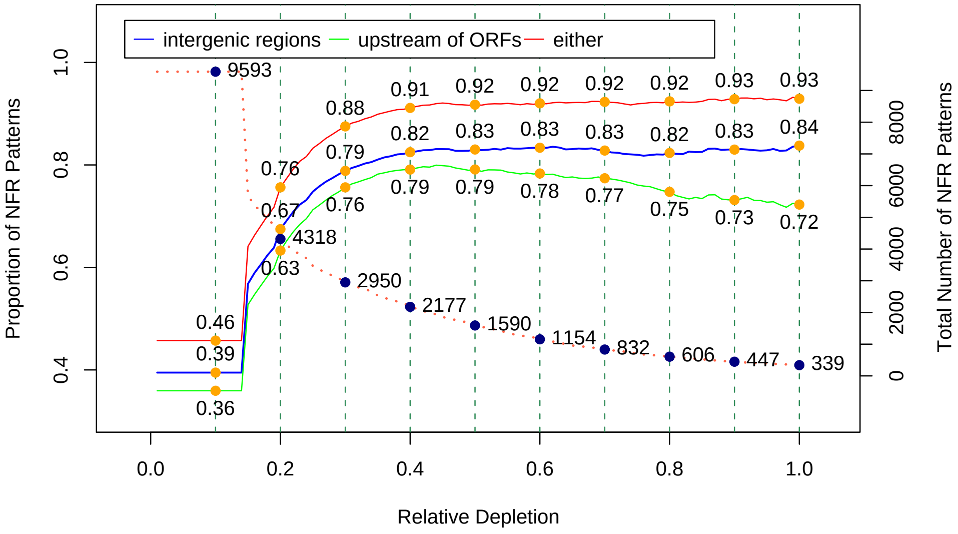

Supplementary Figure 5: Locations of NFRs vs. relative depletion

The proportions of NFR patterns located in intergenic regions, 500bp upstream of ORFs, and either intergenic or 500bp upstream region according to different cutoffs of relative depletion. The dash line indicates the total number of NFR patterns at different cutoffs of relative depletion (corresponding to the axis on the right side).

Among all 6640 intergenic regions, 1617 (24.4%) are divergent intergenic regions, 3087 (46.5%) are tandem intergenic regions, and 1599 (24.1%) are convergent intergenic regions. We excluded 337 (5%) intergenic regions, in which at least one of the adjacent chromosomal features lacks transcription orientation, e.g., ARS. The total lengths of divergent, tandem, and convergent intergenic regions are 0.47Mb (17% of all the intergenic regions), 1.42Mb (51%), and 0.87Mb (31%) respectively.

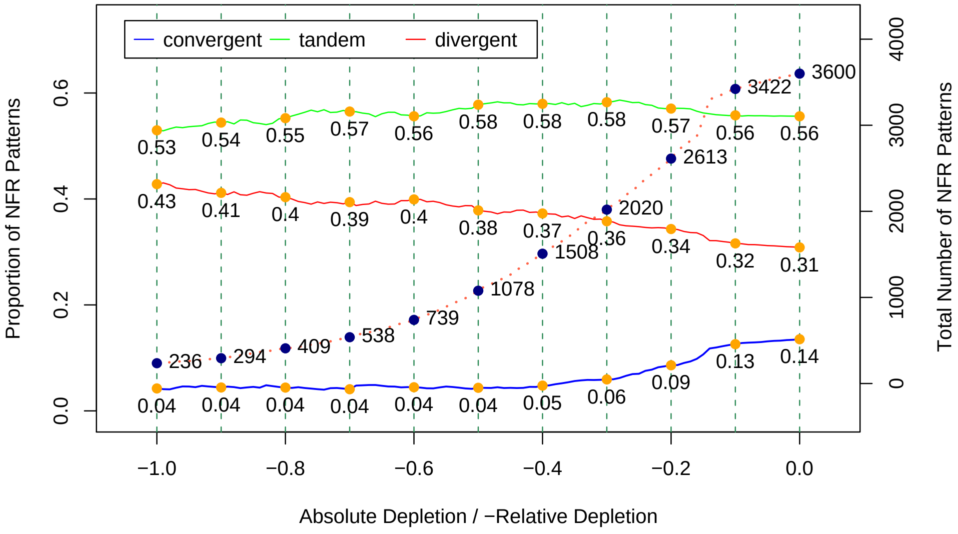

Supplementary Figure 6: Different intergenic regions vs. NFR absolute/relative depletion

The proportions of NFRs located at convergent, divergent and tandom intergenic regions according to different cutoffs of relative depletion and absolute depletion. Specifically, a cutoff α ($\alpha < 0$) indicates the absolute depletion is smaller than α and the relative depletion is bigger than $-\alpha$. The dash line indicates the total number of NFRs within the three types of intergenic regions at different cutoffs (corresponding to the axis on the right side).

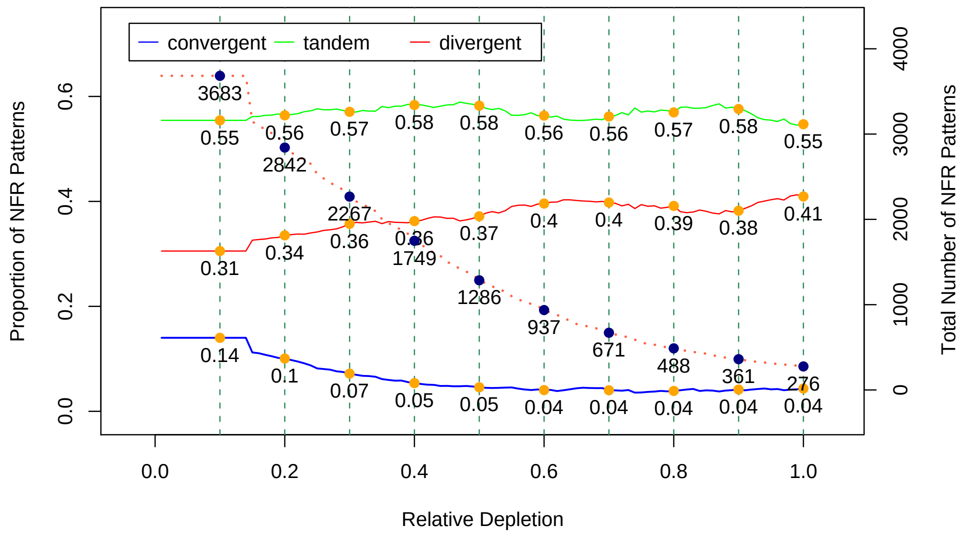

Supplementary Figure 7: Different intergenic regions vs. NFR absolute depletion

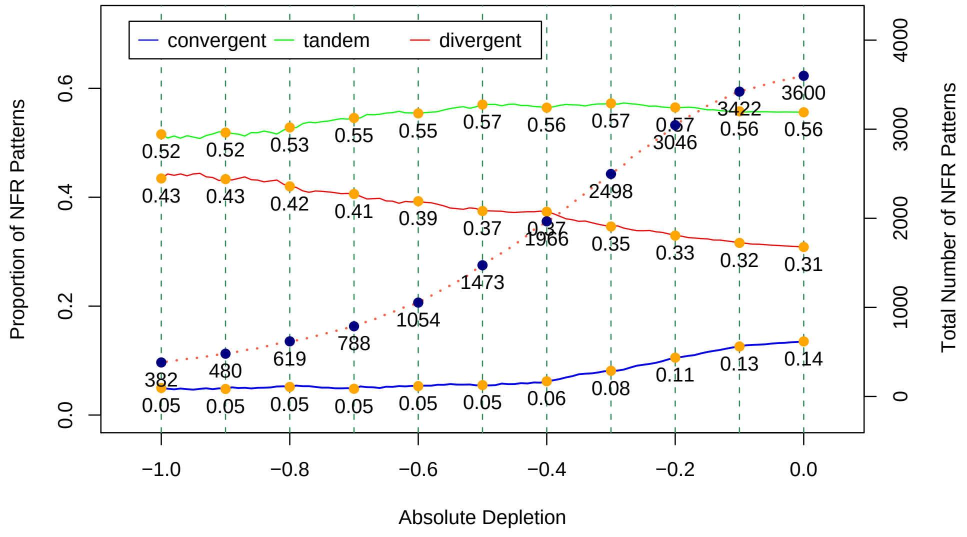

Supplementary Figure 8: Different intergenic regions vs. NFR relative depletion

We also partitioned the promoter regions of 6604 ORFs based on whether they contain TATA box [9]. After excluding 933 promoters whose TATA box information is not available, 1090 (19.2%) of the remaining promoters are classified as TATA box-containing promoters and 4581 (80.8%) are TATA-less promoters. The proportions of NFRs that are located at different promoter regions were examined, and we found that NFRs with heavy nucleosome depletion are more likely to be TATA-containing promoters (Supplementary Figure 9, 10, 11).

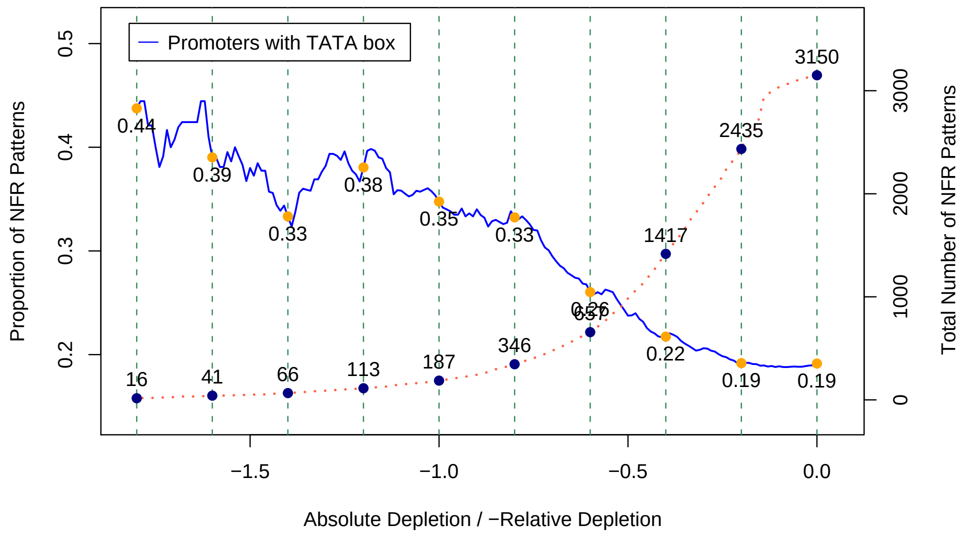

Supplementary Figure 9: TATA box-containing promoters vs. NFR absolute/relative depletion

The proportions of NFR patterns located in 500 bp upstream promoters with TATA box according to different cutoffs of relative depletion and absolute depletion. Specifically, a cutoff α ($\alpha < 0$) indicates the absolute depletion is smaller than α and the relative depletion is bigger than $-\alpha$. The dash line indicates the total number of NFR patterns located in 500 bp upstream promoters at different cutoffs (corresponding to the axis on the right side).

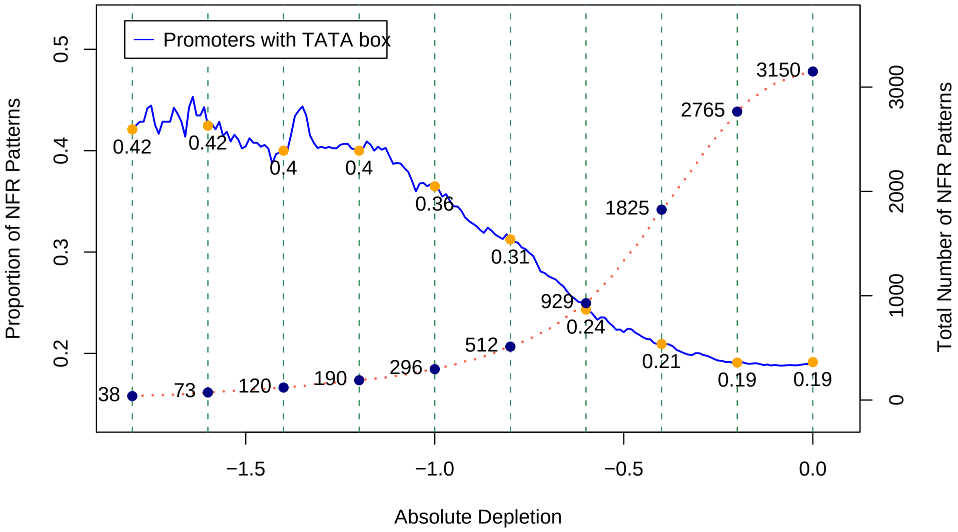

Supplementary Figure 10: TATA box-containing promoters vs. NFR absolute depletion

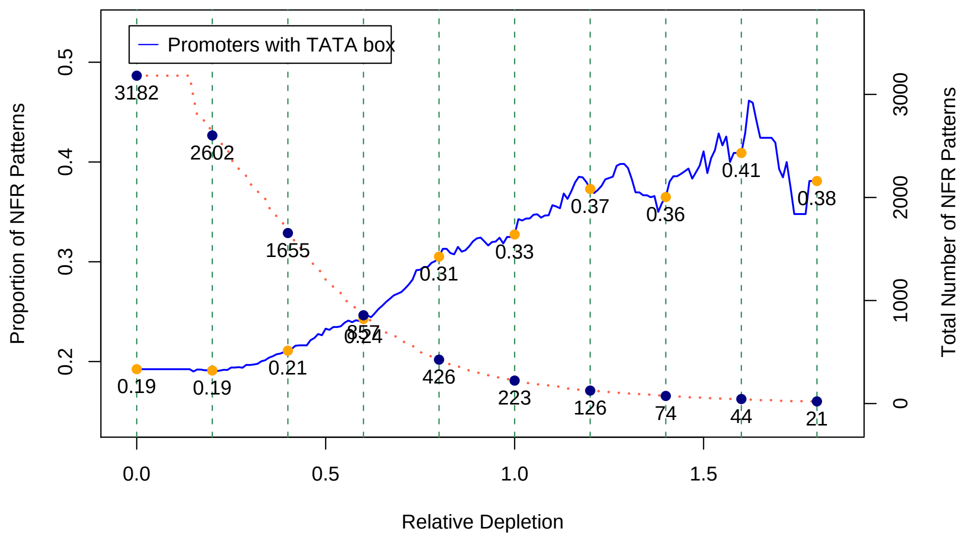

Supplementary Figure 11: TATA box-containing promoters vs. NFR relative depletion

It has been shown previously that TF binding sites are over-represented in nucleosome-depleted promoters [6]. We further examined the co-occurrence of TF binding sites and NFRs in a genome-wide scale. The TF binding sites, including 4312 binding sites with lengths ranging from 4bp to 22bp, were previously inferred by two conservation based motif discovery methods [10] using a genome-wide TF binding study [11]. We define a TF binding site falling into a NFR if the mid-point of the binding site is covered by that NFR. We showed that the proportion of NFRs harboring TF binding sites increases as the degree of nucleosome depletion increase (Supplementary Figure 12, 14, 13).

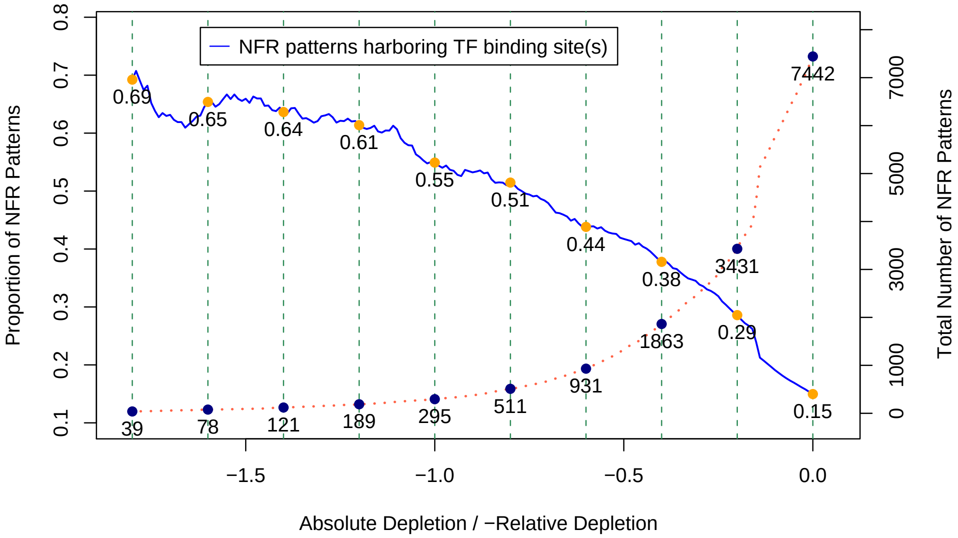

Supplementary Figure 12: TF binding sites vs. absolute/relative depletion
The proportions of NFRs harboring TF binding site(s) according to different cutoffs of relative depletion and absolute depletion. Specifically, a cutoff α ($\alpha < 0$) indicates the absolute depletion is smaller than α and the relative depletion is bigger than $-\alpha$. The dash line indicates the total number of NFR patterns at different cutoffs (corresponding to the axis on the right side).

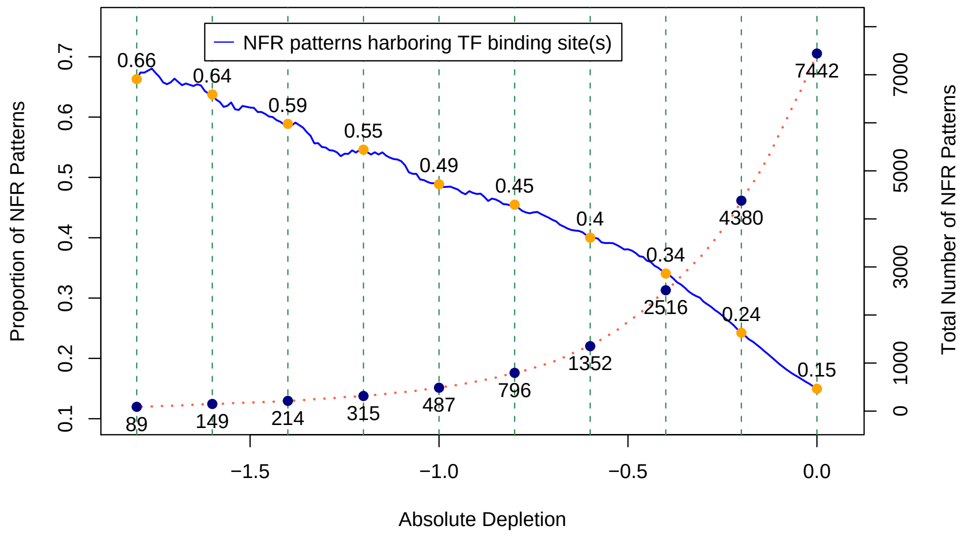

Supplementary Figure 13: TF binding sites vs. absolute depletion

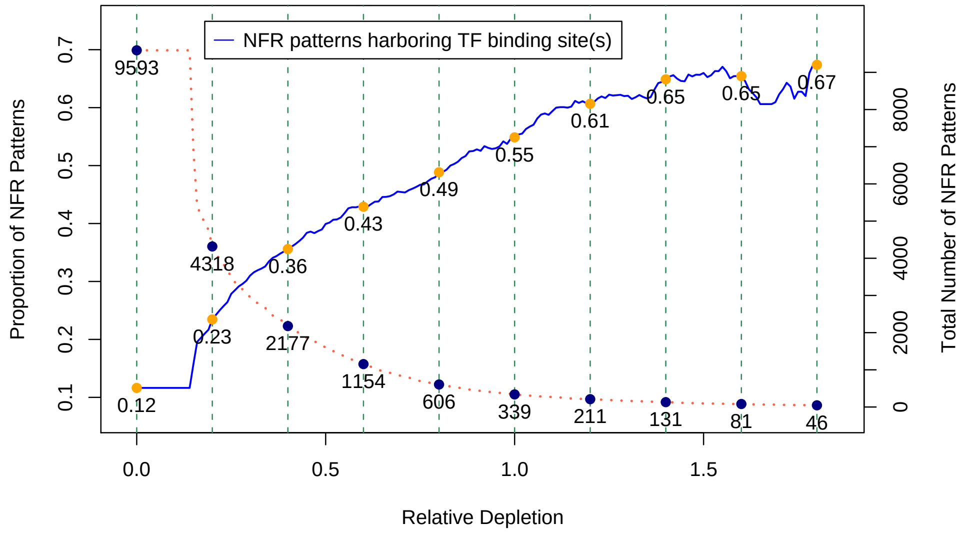

Supplementary Figure 14: TF binding sites vs. relative depletion

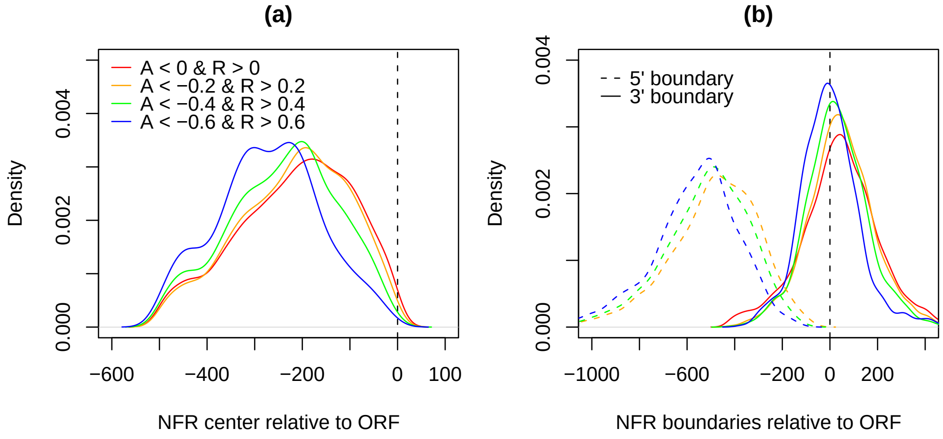

Supplementary Figure 15: Locations of NFRs in the promoters regions relative to ORFs

The locations of 3393 NFRs within the promoter regions (500 upstream of ORFs) relative to the ORF start sites. (a) The centers of NFRs relative to the ORF start sites. (b) The boundaries of NFRs relative to the ORF start sites.

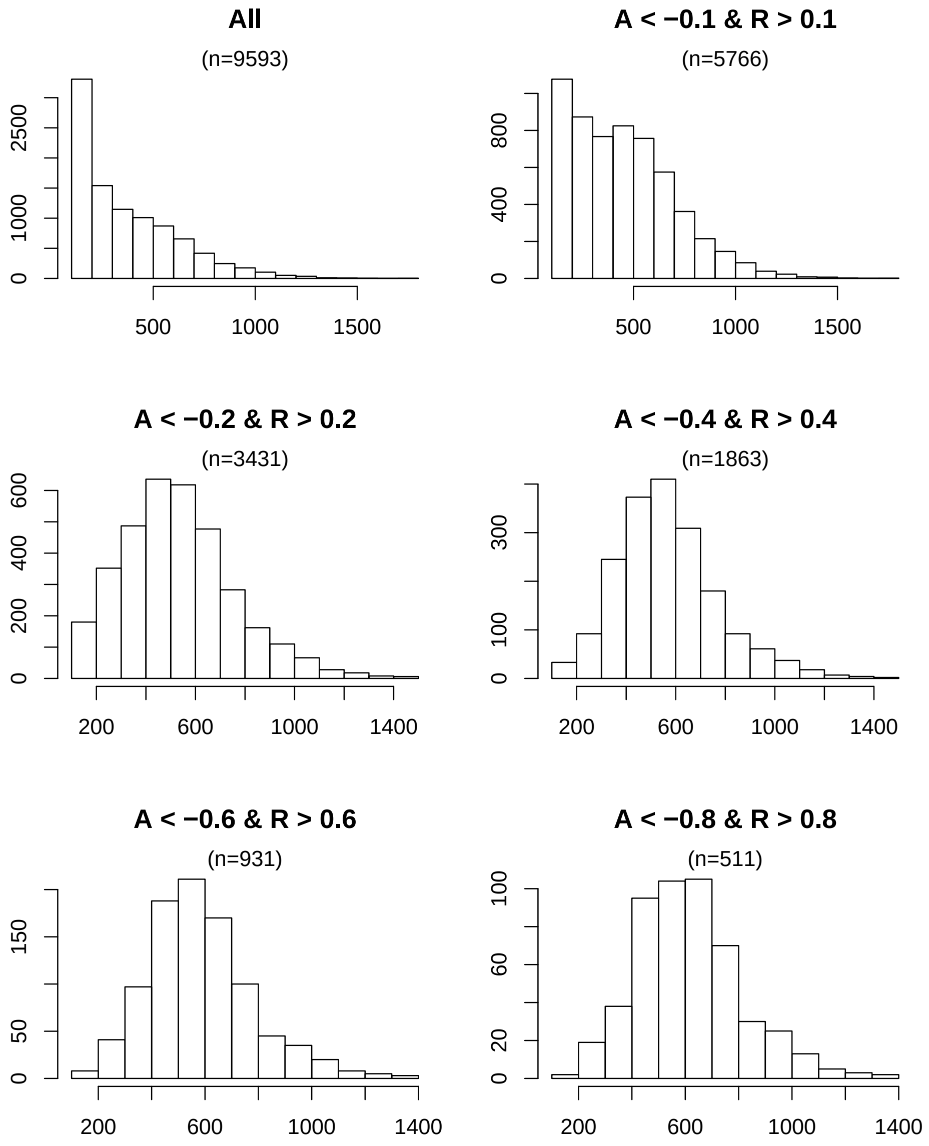

Supplementary Figure 16: NFR lengths vs. absolute/relative depletion
The X-axis indicates the lengths of NFRs in base pair, and the Y-axis indicates the frequency of NFRs. The numbers in parentheses are the counts of NFRs according to the cutoffs of DoND.

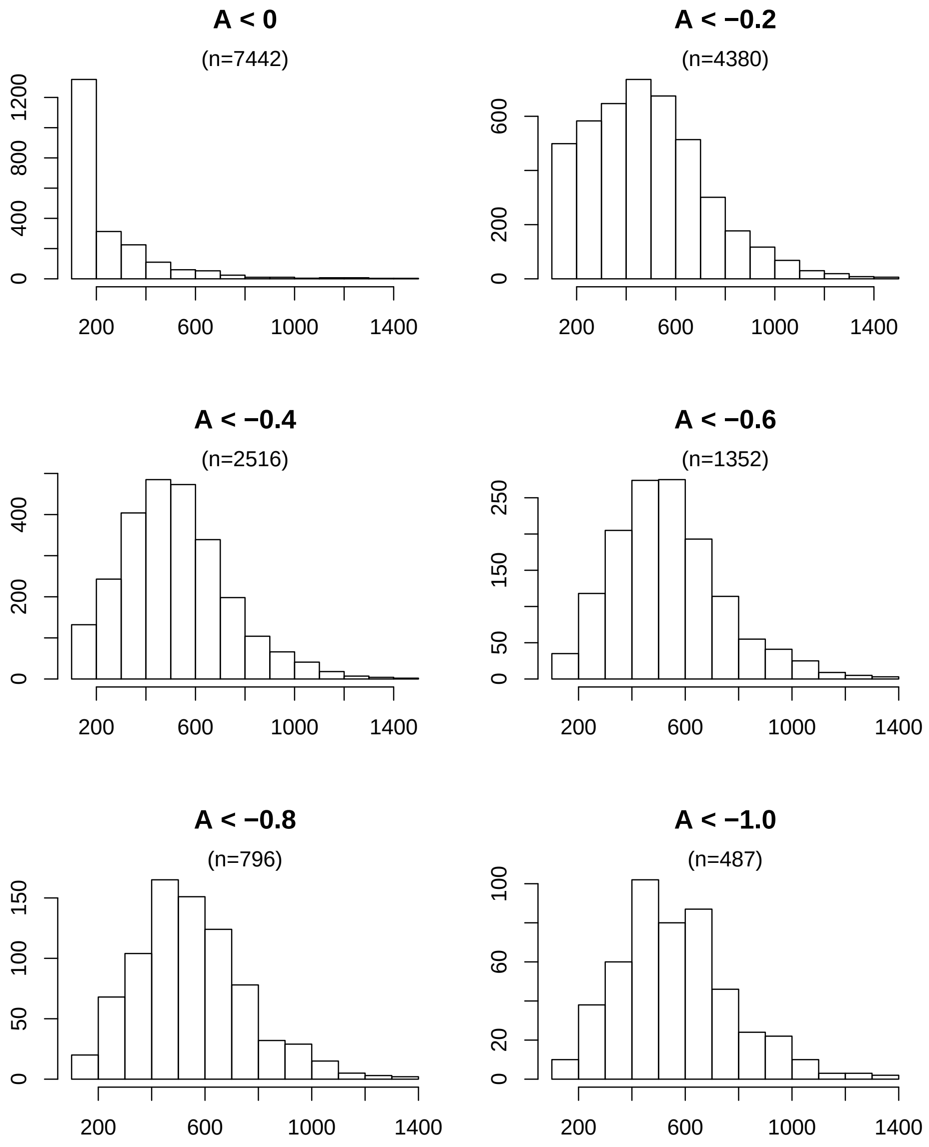

Supplementary Figure 17: NFR lengths vs. absolute depletion

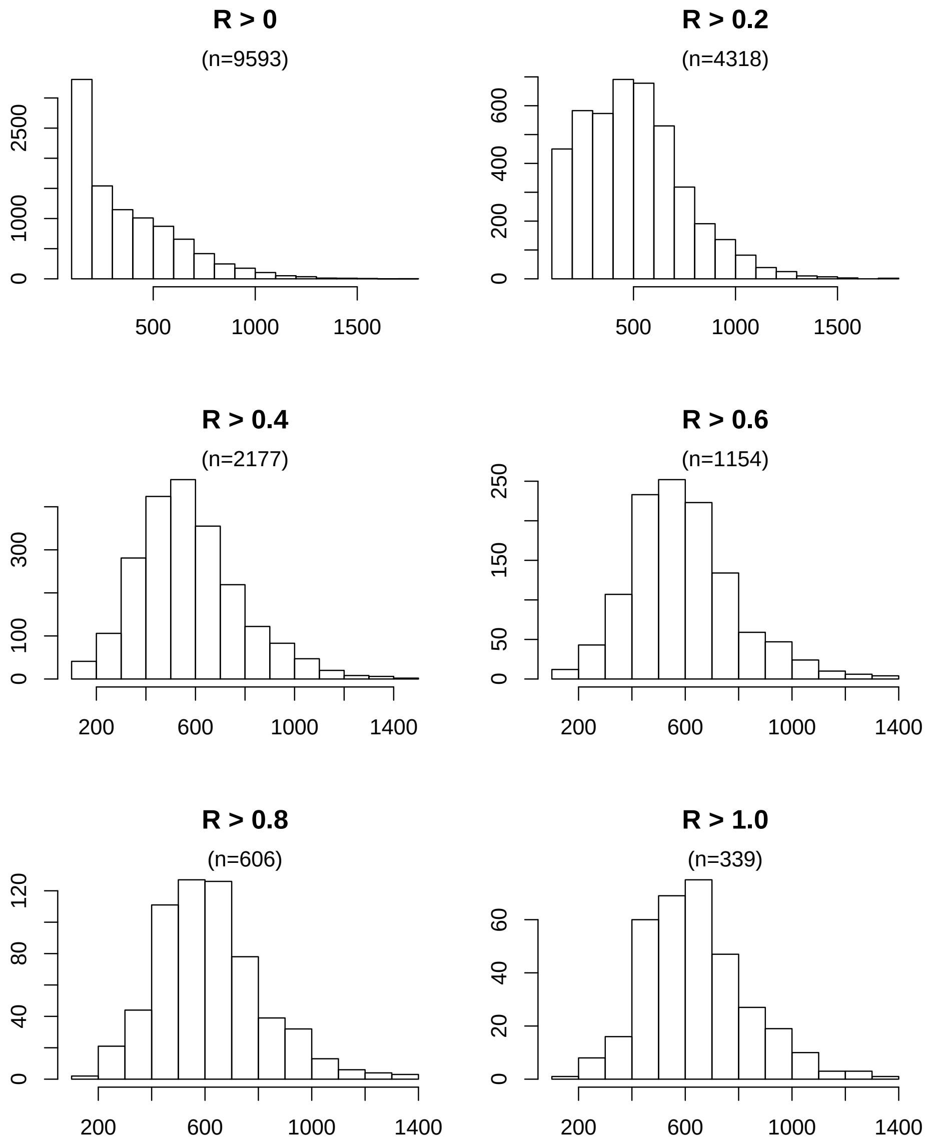

Supplementary Figure 18: NFR lengths vs. relative depletion

6 Nucleosome depletion forces: DNA affinity for histones and transcriptional activity

In order to examine the contributions of transcriptional activity and DNA affinity for histones, we used a bivariate additive linear model with DoND (either absolute depletion or relative depletion) as response and the two factors as covariates. Specifically, the linear model can be written as

$$y = b_0 + b_1 x_1 + b_2 x_2 + e,$$

where y, x_1, and x_2 are DoND, DNA affinity for histones and Pol II binding level respectively, and b_0, b_1, and b_2 are linear regression coefficients and e is the residual error. Because DoND is positively correlated with DNA affinity and negatively correlated with Pol II binding, b_1 is positive and b_2 is negative. The variance explained by DNA affinity and Pol II are $b_1^2 \text{var}(x_1)$ and $b_2^2 \text{var}(x_2)$, respectively. The covariance term is $\text{cov}(b_1 x_1, b_2 x_2) = b_1 b_2 \text{cov}(x_1, x_2)$. Due to the opposite signs of coefficients b_1 and b_2, as well as the weak negative correlation between DNA affinity and Pol II binding level (ranging from -0.088 to 0 in all cases we considered), the covariance term is positive, which we considered as the variance explained by both factors. In addition, since the correlation between x_1 and x_2 is weak, the covariance term is small compared to the total variance of DoND explained by this linear model.

Supplementary Table 5: Correlation matrix of DoND and average Pol II binding level

Variables: A, absolute depletion; R, relative depletion; polNfr, average Pol II binding within NFRs; polPro(1k)/polPro(500), average Pol II binding 1000/500bp upstream of NFRs; polAfr(1k)/polAfr(500), average Pol II binding 1000/500bp downstream of NFRs; polAdj(1k)/polAdj(500), max(polPro, polAfr).

The average Pol II level was calculated using 500bp up- and down-stream of each NFR.

	A	R	polNfr	polPro(1k)	polAfr(1k)	polAdj(1k)
A	1.000	-0.853	-0.086	-0.188	-0.196	-0.342
R	-0.853	1.000	0.042	0.159	0.170	0.282
polNfr	-0.086	0.042	1.000	0.779	0.749	0.824
polPro(1k)	-0.188	0.159	0.779	1.000	0.518	0.813
polAfr(1k)	-0.196	0.170	0.749	0.518	1.000	0.812
polAdj(1k)	-0.342	0.282	0.824	0.813	0.812	1.000

The average Pol II level was calculated using 1000bp up- and down-stream of each NFR.

	A	R	polNfr	polPro(500)	polAfr(500)	polAdj(500)
A	1.000	-0.853	-0.086	-0.176	-0.180	-0.324
R	-0.853	1.000	0.042	0.151	0.158	0.272
polNfr	-0.086	0.042	1.000	0.834	0.816	0.873
polPro(500)	-0.176	0.151	0.834	1.000	0.599	0.847
polAfr(500)	-0.180	0.158	0.816	0.599	1.000	0.841
polAdj(500)	-0.324	0.272	0.873	0.847	0.841	1.000

Supplementary Table 6: Linear model coefficients using all 9593 NFRs

This table lists the coefficients and the corresponding p-values of three linear models:
(1) Absolute depletion $\sim$ DNA,
(2) Absolute depletion $\sim$ Pol II,
(3) Absolute depletion $\sim$ DNA + Pol II.
As we obtained similar results using relative depletion for DoND as those using absolute depletion, and the linear model coefficients are not of main interest, we only list the coefficients using absolute depletion here. The DNA affinity for histones of an NFR was calculated using the average probability of "nucleosome occupancy" inferred by Segal et al. [12] for all the basepairs in the NFR. The average Pol II binding level was calculated respectively for up- and down-stream of each NFR and the maximum of these two averages is defined as the Pol II binding level as described in the paper. The three models were fitted using three different data sets: all 9593 NFRs, 4386 NFRs located in intergenic regions or 500bp upstream of ORFs, and the rest 5207 NFRs.

The average Pol II level was calculated using 500bp up- and down-stream of each NFR.

Model	All NFRs				Intergenic/Upstream				Others			
	DNA		Pol II		DNA		Pol II		DNA		Pol II	
1	0.45	5e-27	NA	NA	1.07	5e-46	NA	NA	0.15	8e-08	NA	NA
2	NA	NA	-0.21	4e-233	NA	NA	-0.23	5e-141	NA	NA	-0.03	7e-09
3	0.4	3e-24	-0.2	2e-230	0.99	3e-45	-0.22	4e-140	0.15	2e-07	-0.03	2e-08

The average Pol II level was calculated using 1000bp up- and down-stream of each NFR.

Model	All NFRs				Intergenic/Upstream				Others			
	DNA		Pol II		DNA		Pol II		DNA		Pol II	
1	0.45	5e-27	NA	NA	1.07	5e-46	NA	NA	0.15	8e-08	NA	NA
2	NA	NA	-0.23	1e-260	NA	NA	-0.26	3e-161	NA	NA	-0.04	1e-09
3	0.4	1e-24	-0.23	3e-258	0.98	7e-45	-0.25	5e-160	0.15	2e-07	-0.04	3e-09

Supplementary Table 7: Linear model coefficients for different intergenic regions

This table lists the coefficients and the corresponding p-values of three linear models:
(1) Absolute depletion ∼ DNA,
(2) Absolute depletion ∼ Pol II,
(3) Absolute depletion ∼ DNA + Pol II.
The three models were fitted using three different data sets: 516 NFRs located in convergent intergenic regions, 2042 NFRs located in tandem intergenic regions, and 1125 NFRs located in divergent intergenic regions.

The average Pol II level was calculated using 500bp up- and down-stream of each NFR.

Model	Convergent				Tandem				Divergent			
	DNA		Pol II		DNA		Pol II		DNA		Pol II	
1	0.8	3e-05	NA	NA	1.14	9e-26	NA	NA	1.08	1e-09	NA	NA
2	NA	NA	-0.11	2e-08	NA	NA	-0.22	4e-69	NA	NA	-0.3	6e-56
3	0.75	5e-05	-0.1	4e-08	1.08	4e-27	-0.22	2e-70	0.85	1e-07	-0.29	6e-54

The average Pol II level was calculated using 1000bp up- and down-stream of each NFR.

Model	Convergent				Tandem				Divergent			
	DNA		Pol II		DNA		Pol II		DNA		Pol II	
1	0.8	3e-05	NA	NA	1.14	9e-26	NA	NA	1.08	1e-09	NA	NA
2	NA	NA	-0.11	1e-07	NA	NA	-0.25	4e-73	NA	NA	-0.34	6e-66
3	0.75	5e-05	-0.11	2e-07	1.08	4e-27	-0.24	2e-74	0.81	3e-07	-0.33	2e-63

Supplementary Table 8: Linear model coefficients for different promoter regions

This table lists the coefficients and the corresponding p-values of three linear models:
(1) Absolute depletion ∼ DNA,
(2) Absolute depletion ∼ Pol II,
(3) Absolute depletion ∼ DNA + Pol II.
The three models were fitted using two different data sets: 2570 NFRs located in TATA-less promoter regions, and 612 NFRs located in the promoter regions with TATA box.

The average Pol II level was calculated using 500bp up- and down-stream of each NFR.

Model	TATA-less				TATA			
	DNA		Pol II		DNA		Pol II	
1	0.54	2e-09	NA	NA	1.14	2e-09	NA	NA
2	NA	NA	-0.25	2e-117	NA	NA	-0.27	2e-26
3	0.42	3e-07	-0.24	3e-115	1.16	2e-11	-0.27	2e-28

The average Pol II level was calculated using 1000bp up- and down-stream of each NFR.

Model	TATA-less				TATA			
	DNA		Pol II		DNA		Pol II	
1	0.54	2e-09	NA	NA	1.14	2e-09	NA	NA
2	NA	NA	-0.27	2e-126	NA	NA	-0.3	5e-31
3	0.42	2e-07	-0.27	2e-124	1.13	2e-11	-0.3	8e-33

Supplementary Table 9: Linear model coefficients for TFBS-containing or TFBS-less NFRs

This table lists the coefficients and the corresponding p-values of three linear models:
(1) Absolute depletion $\sim$ DNA,
(2) Absolute depletion $\sim$ Pol II,
(3) Absolute depletion $\sim$ DNA + Pol II.
The three models are fitted using two different data sets: 1116 NFRs harboring TFBSs, and 8477 NFRs that do not contain TFBSs (TFBS-less).

The average Pol II level was calculated using 500bp up- and down-stream of each NFR.

Model	TFBS-less				TFBS			
	DNA		Pol II		DNA		Pol II	
1	1.97	7e-28	NA	NA	0.21	1e-08	NA	NA
2	NA	NA	-0.24	2e-24	NA	NA	-0.14	6e-135
3	1.85	1e-26	-0.22	3e-23	0.19	8e-08	-0.14	4e-134

The average Pol II level was calculated using 1000bp up- and down-stream of each NFR.

Model	TFBS-less				TFBS			
	DNA		Pol II		DNA		Pol II	
1	1.97	7e-28	NA	NA	0.21	1e-08	NA	NA
2	NA	NA	-0.28	3e-30	NA	NA	-0.16	7e-151
3	1.83	8e-27	-0.26	3e-29	0.19	5e-08	-0.16	3e-150

Supplementary Table 10: Compare the effects of DNA affinity for histones and transcriptional activity using absolute depletion for DoND

We compared the effects of DNA affinity for histones and transcriptional activity by the following three linear models:

(1) Absolute depletion $\sim$ DNA,

(2) Absolute depletion $\sim$ Pol II,

(3) Absolute depletion $\sim$ DNA + Pol II,

where Pol II signal was calculated as the maximum of the two averages across 1000bp up- and down-stream of each NFR.

Column "N" refers to the number of NFRs. R_1^2, R_2^2, and R_3^2 denotes the R^2 of model (1), (2), and (3) respectively. $R^2_{Total} = R^2_3$, which is the total proportion of variance explained by DNA affinity for histones or transcriptional activity. $R^2_{DNA} = R^2_3 - R^2_2$, which is the R^2 explained solely by DNA affinity for histones. $R^2_{PolII} = R^2_3 - R^2_1$, which is the R^2 explained solely by Polymerase II binding signal. $R^2_{Both} = R^2_{Total}$ - R^2_{PolII} - R^2_{DNA}, denoting the R^2 explained by both Polymerase II signal and DNA affinity for histones. R^2_{Both} is not zero due to the correlation between DNA affinity and Polymerase II binding [13]. P_{PolII} is the ANOVA p-value comparing model (3) against model (1). P_{DNA} is the ANOVA p-value comparing model (3) against model (2).

	N	R^2_{Total}	R^2_{DNA}	R^2_{PolII}	R^2_{Both}	P_{PolII}	P_{DNA}
All NFRs	9593	0.1263	0.0096(7.6%)	0.1142(90.4%)	0.0024(1.9%)	3e-258	1e-24
Inter./Up.	4386	0.1911	0.0373(19.5%)	0.1459(76.3%)	0.0079(4.1%)	5e-160	7e-45
Others	5207	0.0122	0.0052(42.6%)	0.0067(54.9%)	3e-04(2.5%)	3e-09	2e-07
Convergent	516	0.0837	0.0302(36.1%)	0.0499(59.6%)	0.0036(4.3%)	2e-07	5e-05
Tandem	2042	0.1955	0.0472(24.1%)	0.1429(73.1%)	0.0053(2.7%)	2e-74	4e-27
Divergent	1125	0.2483	0.0179(7.2%)	0.2159(87.0%)	0.0146(5.9%)	2e-63	3e-07
TATA	612	0.2542	0.0567(22.3%)	0.1965(77.3%)	9e-04(0.4%)	8e-33	2e-11
TATA-less	2570	0.2082	0.0084(4.0%)	0.1941(93.2%)	0.0057(2.7%)	2e-124	2e-07
TFBS	1116	0.198	0.0874(44.1%)	0.0961(48.5%)	0.0146(7.4%)	3e-29	8e-27
TFBS-less	8477	0.0808	0.0032(4.0%)	0.077(95.3%)	6e-04(0.7%)	3e-150	5e-08

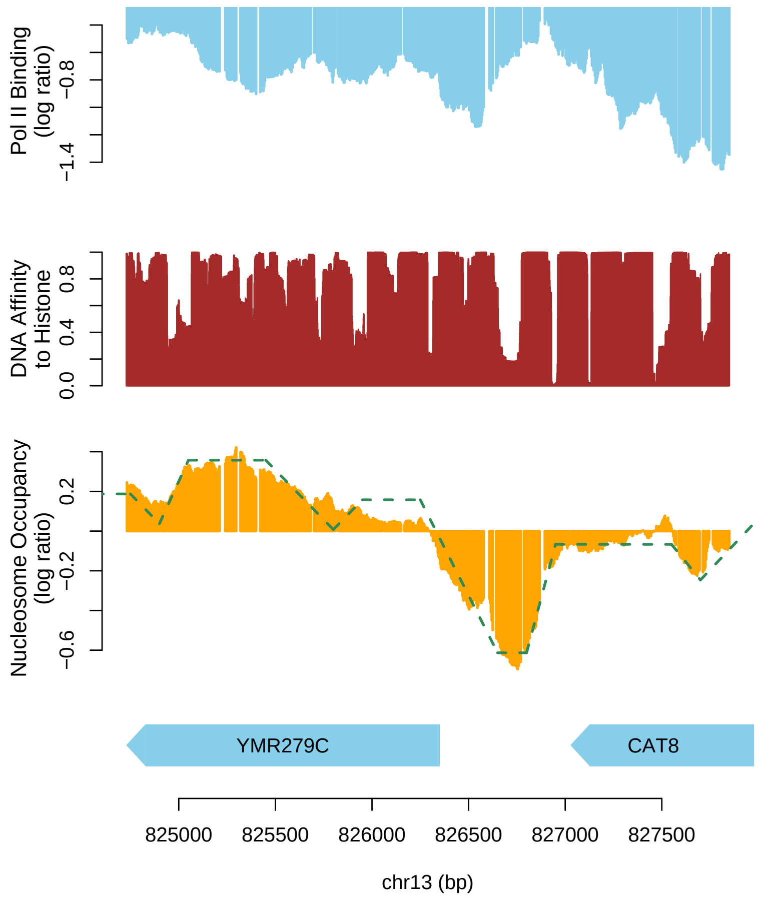

Supplementary Figure 19: This figure shows the Pol II binding level (log ratio from our ChIP-chip results), DNA affinity for histones (posterior probability of histone binding from Segal et al. [12]), and nucleosome occupancy level (log ratio from our ChIP-chip results) around the gene YMR279C.

Supplementary Table 11: Compare the effects of DNA affinity for histones and transcriptional activity using relative depletion for DoND.

	N	R^2_{Total}	R^2_{DNA}	R^2_{PolII}	R^2_{Both}	P_{PolII}	P_{DNA}
All NFRs	9593	0.0887	0.009(10.1%)	0.0779(87.8%)	0.0019(2.1%)	5e-173	3e-22
Inter./Up.	4386	0.1203	0.0293(24.4%)	0.0857(71.2%)	0.0054(4.5%)	1e-90	5e-33
Others	5207	0.0062	0.005(80.6%)	0.001(16.1%)	1e-04(1.6%)	0.02	3e-07
Convergent	516	0.0428	0.0337(78.7%)	0.0076(17.8%)	0.0015(3.5%)	0.04	3e-05
Tandem	2042	0.1081	0.0264(24.4%)	0.0787(72.8%)	0.003(2.8%)	2e-39	1e-14
Divergent	1125	0.1731	0.0156(9.0%)	0.1465(84.6%)	0.011(6.4%)	1e-41	5e-06
TATA	612	0.1749	0.0423(24.2%)	0.132(75.5%)	7e-04(0.4%)	2e-21	3e-08
TATA-less	2570	0.1204	0.007(5.8%)	0.1095(90.9%)	0.0039(3.2%)	2e-67	6e-06
TFBS	1116	0.1483	0.0668(45.0%)	0.0707(47.7%)	0.0109(7.3%)	5e-21	5e-20
TFBS-less	8477	0.0464	0.0029(6.2%)	0.0431(92.9%)	4e-04(0.9%)	2e-83	4e-07

7 NFRs outside of the promoters or intergenic regions

Supplementary Table 12: Distribution of 145 NFRs falling outside of the promoters or intergenic regions.

This table lists 145 NFRs with high DoND ($R > 0.4$ and $A < -0.4$) that lie outside of intergenic or 500bp upstream of ORFs.

Feature	Number of NFRs
ORF	58
tRNA	52
ARS	16
long terminal repeat	9
Y' element	4
intron	2
rRNA	2
X element core sequence	1
snRNA	1

Supplementary Table 13: List of 25 verified ORFs containing genic NFRs.

ORF	Symbol	Chr	Start	End
YLL042C	ATG10	12	52589	52086
YPL111W	CAR1	16	339943	340944
YIR030C	DCG1	9	412767	412033
YPR166C	MRP2	16	876625	876278
YFL003C	MSH4	6	137152	134516
YHR091C	MSR1	8	286772	284841
YAR002W	NUP60	1	152259	153878
YKR003W	OSH6	11	445024	446370
YDL232W	OST4	4	38488	38598
YLR148W	PEP3	12	434642	437398
YFR034C	PHO4	6	225946	225008
YOR361C	PRT1	15	1017650	1015359
YGR170W	PSD2	7	837147	840563
YOR348C	PUT4	15	988779	986896
YOR210W	RPB10	15	738321	738533
YLR141W	RRN5	12	423684	424775
YOL110W	SHR5	15	109176	109889
YOL122C	SMF1	15	91419	89692
YDR308C	SRB7	4	1078445	1078023
YBR150C	TBS1	2	544487	541203
YNL070W	TOM7	14	493367	493549
YER093C	TSC11	5	347608	343316
YLR024C	UBR2	12	193282	187664
YAL002W	VPS8	1	143709	147533
YAR035W	YAT1	1	190187	192250

Supplementary Table 14: List of 33 dubious or uncharacterized ORFs containing genic NFRs. The dubious ORFs, but not the uncharacterized ORFs, are over-represented in ORFs containing genic NFRs (hypergeometric p-value = 1e-5) based on the SGD [8] annotations, implying that some of the dubious ORFs may not be real coding genes.

Type	ORF	Symbol	Chr	Start	End	Strand
Dubious	YAR060C	NA	1	217483	217148	C
Dubious	YBL048W	NA	2	127302	127613	W
Dubious	YBR209W	NA	2	642578	642895	W
Dubious	YDR010C	NA	4	465380	465048	C
Dubious	YDR215C	NA	4	894498	894115	C
Dubious	YDR274C	NA	4	1011956	1011585	C
Dubious	YDR278C	NA	4	1017314	1016997	C
Dubious	YGR107W	NA	7	702671	703120	W
Dubious	YHL041W	NA	8	17390	17839	W
Dubious	YHR070C-A	NA	8	236514	236104	C
Dubious	YHR212C	NA	8	538094	537759	C
Dubious	YIL054W	NA	9	254541	254858	W
Dubious	YKL102C	NA	11	248011	247706	C
Dubious	YML089C	NA	13	91409	91041	C
Dubious	YML122C	NA	13	26419	26039	C
Dubious	YNL285W	NA	14	96173	96544	W
Dubious	YOR029W	NA	15	384600	384935	W
Dubious	YOR050C	NA	15	424619	424272	C
Dubious	YOR343C	NA	15	968471	968145	C
Dubious	YPR014C	NA	16	587515	587186	C
Dubious	YPR064W	NA	16	678948	679367	W
Uncharacterized	YAR064W	NA	1	220189	220488	W
Uncharacterized	YBL044W	NA	2	136001	136369	W
Uncharacterized	YER077C	NA	5	316596	314530	C
Uncharacterized	YFR032C-B	NA	6	223961	223698	C
Uncharacterized	YGL176C	NA	7	173085	171421	C
Uncharacterized	YGR068C	NA	7	627088	625328	C
Uncharacterized	YHR202W	NA	8	502388	504196	W
Uncharacterized	YHR213W-B	NA	8	540800	541099	W
Uncharacterized	YJR003C	NA	10	442468	440909	C
Uncharacterized	YMR196W	NA	13	655075	658341	W
Uncharacterized	YOR268C	NA	15	825931	825533	C
Uncharacterized	YPR159C-A	NA	16	860411	860310	C

References

[1] Pokholok DK, Harbison CT, Levine S, Cole M, Hannett NM, Lee TI, Bell GW, Walker K, Rolfe PA, Herbolsheimer E, Zeitlinger J, Lewitter F, Gifford DK, Young RA: **Genome-wide map of nucleosome acetylation and methylation in yeast**. *Cell* 2005, **122**(4):517–527.

[2] Heintzman N, Stuart R, Hon G, Fu Y, Ching C, Hawkins R, Barrera L, Van S Calcar, Qu C, Ching K, Wang W, Weng Z, Green R, Crawford G, Ren B: **Distinct and predictive chromatin signatures of transcriptional promoters and enhancers in the human genome**. *Nat Genet* 2007, **39**:311–8.

[3] Rabiner L: **A tutorial on hidden Markov models and selected applications in speech recognition**. *Proceedings of the IEEE* 1989, **77**(2):257–286.

[4] Ostendorf M, Digalakis V, Kimball O: **From HMM's to segment models: a unified view of stochastic modelingfor speech recognition**. *IEEE Transactions on Speech and Audio Processing* 1996, **4**(5):360–378.

[5] Durbin R, Eddy SR, Krogh A, Mitchison G: *Biological Sequence Analysis: Probabilistic Models of Proteins and Nucleic Acids*. Cambridge University Press 1998.

[6] Bernstein BE, Liu CL, Humphrey EL, Perlstein EO, Schreiber SL: **Global nucleosome occupancy in yeast**. *Genome Biol* 2004, **5**(9):R62.

[7] Lee CK, Shibata Y, Rao B, Strahl BD, Lieb JD: **Evidence for nucleosome depletion at active regulatory regions genome-wide**. *Nat Genet* 2004, **36**(8):900–5.

[8] Cherry JM, Adler C, Ball C, Chervitz SA, Dwight SS, Hester ET, Jia Y, Juvik G, Roe T, Schroeder M, Weng S, Botstein D: **SGD: Saccharomyces Genome Database**. *Nucleic Acids Res* 1998, **26**:73–79.

[9] Basehoar AD, Zanton SJ, Pugh BF: **Identification and distinct regulation of yeast TATA box-containing genes**. *Cell* 2004, **116**(5):699–709.

[10] MacIsaac KD, Wang T, Gordon DB, Gifford DK, Stormo GD, Fraenkel E: **An improved map of conserved regulatory sites for Saccharomyces cerevisiae**. *BMC Bioinformatics* 2006, **7**:113.

[11] Harbison CT, Gordon DB, Lee TI, Rinaldi NJ, Macisaac KD, Danford TW, Hannett NM, Tagne JB, Reynolds DB, Yoo J, Jennings EG, Zeitlinger J, Pokholok DK, Kellis M, Rolfe PA, Takusagawa KT, Lander ES, Gifford DK, Fraenkel E, Young RA: **Transcriptional regulatory code of a eukaryotic genome**. *Nature* 2004, **431**(7004):99–104.

[12] Segal E, Fondufe-Mittendorf Y, Chen L, Thastrom A, Field Y, Moore IK, Wang JP, Widom J: **A genomic code for nucleosome positioning**. *Nature* 2006, **442**(7104):772–8.

[13] Nachtsheim CJ, Neter J, Kutner MH: *Applied linear statistical models.* McGraw-Hill 2004.
